# Acquiring of photosensitivity by *Mycobacterium tuberculosis *in vitro and inside infected macrophages is associated with accumulation of endogenous Zn–porphyrins

**DOI:** 10.1038/s41598-024-51227-z

**Published:** 2024-01-08

**Authors:** Margarita O. Shleeva, Irina A. Linge, Ivan A. Gligonov, Galina N. Vostroknutova, Denis M. Shashin, Andrey M. Tsedilin, Alexander S. Apt, Arseny S. Kaprelyants, Alexander P. Savitsky

**Affiliations:** 1 A.N. Bach Institute of Biochemistry, Federal Research Centre ‘Fundamentals of Biotechnology’ of the Russian Academy of Sciences, Moscow, Russia; 2 Laboratory for Immunogenetics, Central Tuberculosis Research Institute, Moscow, Russia

**Keywords:** Biophysical chemistry, Tuberculosis, Biological fluorescence, Biophysical chemistry, Pathogens

## Abstract

*Mycobacterium tuberculosis* (*Mtb*) is able to transition into a dormant state, causing the latent state of tuberculosis. Dormant mycobacteria acquire resistance to all known antibacterial drugs and can survive in the human body for decades before becoming active. In the dormant forms of *M. tuberculosis,* the synthesis of porphyrins and its Zn-complexes significantly increased when 5-aminolevulinic acid (ALA) was added to the growth medium. Transcriptome analysis revealed an activation of 8 genes involved in the metabolism of tetrapyrroles during the *Mtb* transition into a dormant state, which may lead to the observed accumulation of free porphyrins. Dormant *Mtb* viability was reduced by more than 99.99% under illumination for 30 min (300 J/cm^2^) with 565 nm light that correspond for Zn–porphyrin and coproporphyrin absorptions. We did not observe any PDI effect in vitro using active bacteria grown without ALA. However, after accumulation of active cells in lung macrophages and their persistence within macrophages for several days in the presence of ALA, a significant sensitivity of active *Mtb* cells (ca. 99.99%) to light exposure was developed. These findings create a perspective for the treatment of latent and multidrug-resistant tuberculosis by the eradication of the pathogen in order to prevent recurrence of this disease.

## Introduction

Currently, antituberculosis treatment in the clinic faces significant problems due to spreading antibiotic-resistant strains of the pathogen. In addition to this genetically determined resistance, there is a resistance arising from physiological plasticity of mycobacteria. Thus, about one-quarter of the world's population has tuberculosis infection in the latent form, which is caused by the transition of the pathogen into a state of dormancy. Dormant cells of mycobacteria are characterized by low metabolic activity and the absence of syntheses of macromolecules and the cell wall. The known anti-tuberculosis drugs applicable in medical practice are not active against dormant mycobacteria. In recent years, it has been demonstrated that infection with COVID-19 often results in the transition of latent tuberculosis into an active form, which in a significant percentage of cases turns out to be drug-resistant^1^. This situation calls for a search for and development of new approaches designed to combat resistant forms of infection^2^.

Antimicrobial photodynamic inactivation (aPDI) is one of the alternatives to the antibiotic approach that is able to inactivate bacteria, including antibiotic-resistant forms. Antibiotics and aPDI can act synergistically^3^.

One of the most effective photosensitizers are porphyrins^4^, among which zinc complexes standout^5–11^. Zn–coproporphyrin is considered as a promising compound for PDT of tumors^12^.

The antimicrobial effect of aPDI by exogenic photosensitizers is limited by the ability of photosensitizers to interact with bacteria and penetrate through the highly organized outer membrane of microorganisms^13^, especially mycobacteria.

More promising approach seems to be the usage of endogenous porphyrins, the synthesis of which can be stimulated by adding a precursor of heme synthesis, 5-aminolevulinic acid (ALA), to growing bacteria. ALA can penetrate into bacteria, for example, *Haemophilus parainfluenzae,* through hydrophilic pores present in membranes^14^.

The observed accumulation of endogenous porphyrins during the transition of *Mycolicibacterium (basonym: Mycobacterium) smegmatis* cells into a dormant state^15^ creates the prerequisites for possible aPDI of these bacterial forms. Indeed, we found that illumination of *M.smegmatis* cells led to the inactivation of dormant bacteria, in contrast to the effect of light on vegetative cells taken from logarithmic growth phase^16,17^.

However, neither exploitation of endogenous PSs nor their ALA-mediated induction for aPDI has been studied in relation to *Mycobacterium tuberculosis (Mtb)*.

The aim of this work was to study the possibility of photoinactivation of vegetative and dormant forms of *Mtb* using endogenous porphyrins.

In this study, we report on significant accumulation of porphyrins and Zn porphyrins in *Mtb* cells after growth in the presence of ALA for vegetative and dormant states which results in very high sensitivity of bacteria in both physiological states to photoinactivation in vitro and ex vivo in macrophages.

## Results

### ALA-mediated stimulation of porphyrin synthesis in dormant and vegetative cells of *M. tuberculosis*

In the present study, dormant forms of *Mtb* were obtained as a result of gradual environmental acidification in the late stationary phase (40–60 days from the moment of inoculation) under aerobic conditions^18^. Such dormant forms were characterized by morphological, biochemical and physiological changes in comparison with actively grown bacteria^18^ as well as by differences on transcriptomic and proteomic level^19,20^. The appearance of dormant forms was monitored by the decrease in uracil incorporation in the culture, as well as the development of “non-culturability” (decrease in CFU number up to zero^18^). However, dormant “non-culturable” cells are able for resuscitation in liquid medium as shown elsewhere^18,21^. Therefore, an alternative to CFU, the MPN assay of viability (based on cell cultivation in liquid medium)^18,21^ was used in all experiments below.

**Figure 1:**
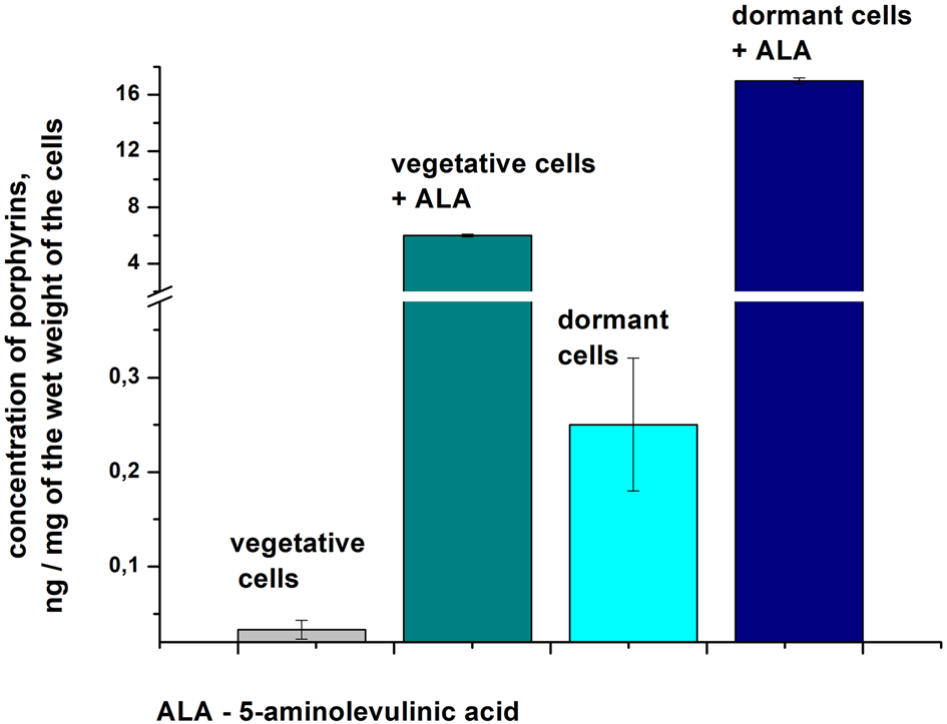
5-aminolevulenic acid (ALA) stimulates the synthesis of endogenous porphyrins in *M. tuberculosis.* Vegetative cells of *M. tuberculosis* cells were grown in Middlebrook 7H9 liquid medium supplemented with ADC and Tween-80 for 14 days (initial bacteria concentration 10^5^ cells per ml). After 5 days post inoculation 3 mM ALA was added to some flasks. Dormant *Mtb* cells were obtained in modified Sauton’s medium as described in “Materials and methods”. ALA (3 mM) was added to dormant culture after 20 days post inoculation. Porphyrin identification in chloroform–methanol extracts was carried out by LC–MS. Bars represented SD from two biological experiments.

As found, dormant forms of *Mtb* contained an elevated (ca. 6 times more) amount of porphyrins in comparison with vegetative cells (0.25 ± 0.05 ng porphyrins/mg wet cell weight in dormant forms versus 0.033 ± 0.01 ng porphyrins/mg wet cell weight in vegetative cells) (Fig. 1).

In order to stimulate the formation of heme synthesis metabolites ALA was added to vegetative *Mtb* cultures and at the stage of transition to the dormant state. A significant number of dormant forms appeared both without and in the presence of 3 mM ALA in the growth medium (Fig. S1). The appearance of dormant forms was monitored by the decrease in uracil incorporation in the culture (Fig. S1a), as well as the development of “non-culturability” (decrease in CFU number up to zero in contrast to high MPN number), (Fig. S1b). The addition of ALA to the model system led to a more rapid development of the medium acidification in the stationary growth phase and an earlier formation of dormant forms (Fig. S1). However, without illumination, in the dark, such a deviation from the usual rate of formation of dormant mycobacteria did not affect the ability of cells obtained under conditions of excess ALA to reactivate judged by MPN assay. Moreover, “+ALA” cells after a long-team period of dormancy (up to 3 years) preserved their potential viability for a longer time in comparison with dormant cells obtained under standard conditions (Fig. S2). Thus, ALA itself does not influence the formation of dormant *Mtb* cells.

It was also found that the addition of ALA to the culture of vegetative *Mtb* 5 days after inoculation did not lead to significant changes in the growth rate of mycobacteria (Fig. S3a).

The maximum production of porphyrins in vegetative *Mtb* cells in the presence of ALA was observed in 10 days after the addition of ALA to the culture, but the amount of porphyrins formed in vegetative *Mtb* was much less than in dormant mycobacteria (Fig. 1).

An overall increase in the total amount of all porphyrins in dormant *Mtb* cells formed with ALA was 85 times up to a value of 17 ng/mg wet cell weight (Fig. 1).

The pellet of bacteria grown in the presence of excess ALA differed significantly in color (reddish) from bacteria obtained without ALA (cream). For vegetative and dormant cells, this color was preserved in sediment after extraction with chloroform–methanol–water.

**Figure 2:**
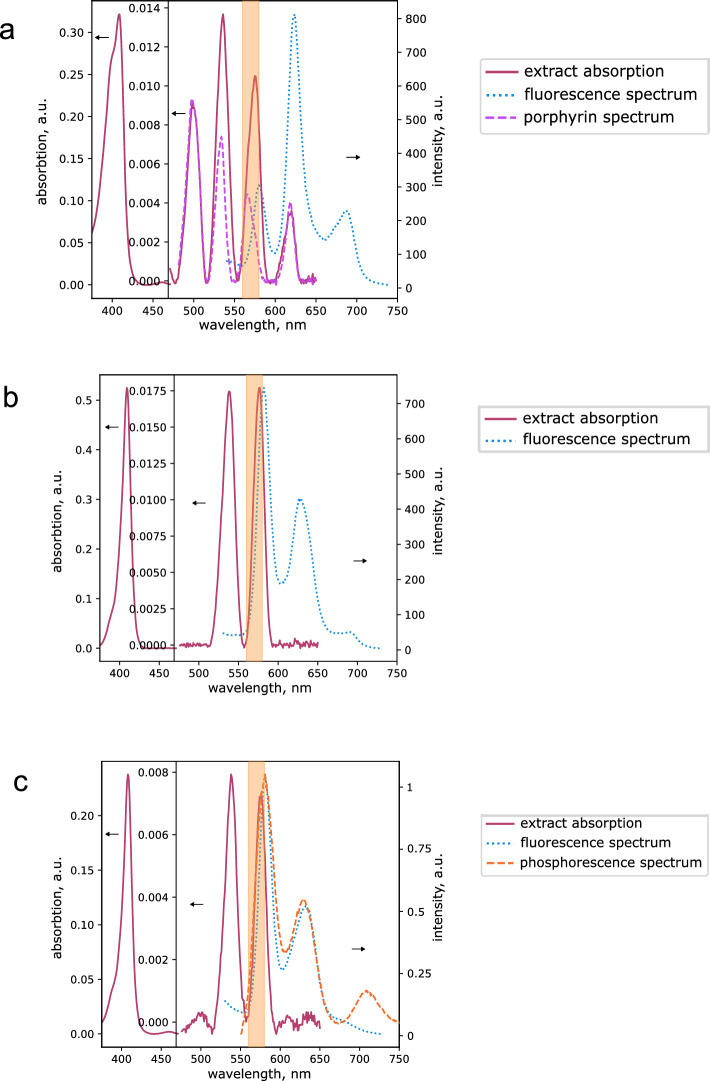
Spectra of the extracts of vegetative and dormant *M. tuberculosis* cells grown in the presence of ALA. *Mtb* cells were inoculated in Middlebrook 7H9 liquid medium supplemented by ADC, Tween-80, and 3 mM ALA at a concentration of 10^5^ cells per ml, followed by incubation at 37 °C, under agitation of 200 rpm. After 20 days, biomass was collected by centrifugation and successively extracted with chloroform–methanol–water and then with 2% Triton × 100 (for details see the “Materials and methods”). Dormant *Mtb* cells were obtained as described in the “Materials and methods”. Then, 3 mM of ALA was added after 20 days post inoculation. Sixty-day-old dormant mycobacteria were harvested and porphyrins were extracted. The orange area corresponds to the illumination at 565/24 nm. (**A**) Absorption and fluorescence spectra chloroform–methanol-water extract of vegetative *Mtb* cells grown in the presence of ALA. Mostly free porphyrin was extracted with a certain amount of Zn porphyrin. (**B**) Absorption and fluorescence spectra at 410 nm excitation of 2% Triton X-100 extract of vegetative *Mtb* cells grown in the presence of ALA. Preferential extraction of Zn porphyrin with a small admixture of free porphyrin. (**C**) Absorption, fluorescence, and phosphorescence spectra at 410 nm excitation of 2% Triton × 100 extract of dormant *Mtb* cells grown in the presence of ALA. Preferential extraction of Zn porphyrin with a small admixture of free porphyrin. Experiments were repeated three times with similar results.

The absorption spectra of the chloroform–methanol extract of the vegetative *Mtb* cells grown in the presence of the ALA clearly indicated in the visible area the superposition of four-band spectra of the absorption of free (without metal) porphyrins (maxima 500, 540, 575 and 620 nm) and two-band spectrum characteristic of Zn–porphyrin (maxima at 545 and 575 nm) (Fig. 2a). The difference between spectra of chlorophorm-methanol extract and spectra of pure coproporphyrin III in chlorophorm-methanol normalized to the absorbance at 500 nm practically coincides with the spectrum of pure Zn–coproporphyrin in the same solvent (Fig. S4).

The fluorescence spectrum of the extract also corresponds to the fluorescence of the mixture of free porphyrin (maxima 620 and 685 nm) and Zn–porphyrin (the main maximum of 580 nm) (Fig. 2a).

The fluorescence spectra of "Triton" extracts also revealed the characteristic maxima at 620 and 680 nm typical for free porphyrins and a strong increase in the maximum at 580 nm typical for the Zn–porphyrin (Fig. 2b, Fig. S4). The appearance of this maximum indicates that Triton X-100 preferably extracts Zn–coproporphyrin derivatives.

In the chloroform–methanol–water extract of dormant *Mtb* forms a low amount of porphyrins was detected. In the “Triton” extract absorption spectrum in the visible range, only a small fraction of four-band spectra of free porphyrin was observed, but pronounced two-band spectra of Zn–porphyrin were detected (Fig. 2c). The “Triton” extract of dormant *Mtb* cells grown in the presence of ALA revealed the fluorescence signal characteristic of the Zn–porphyrin with maxima at 580, 620, and 680 nm (Fig. 2c).

Since Zn–porphyrins have a pronounced phosphorescence and delayed fluorescence at room temperature^22^, we carried out measurements of “Triton” extracts in deoxygenated solutions (Fig. 2c) to prevent quenching of the Zn–porphyrin triplet state. The normalized spectrum showed the presence of a new peak at 710 nm (Fig. 2c, Fig. S5b), characteristic of the triplet state. Since the measured lifetimes of phosphorescence at 710 nm (2.13 ms) and delayed fluorescence at 580 nm (2.25 ms) practically coincide (Fig. S5d), type E delayed fluorescence (thermal activation from the triplet state to the singlet state)^23^ evidently takes place (Fig. S5d).

The fraction of Zn–porphyrins in the sample can be estimated based on the sum of all extracts determined spectrophotometrically in 0.1 N HCl using the extinction coefficients of porphyrin dication given in Falk’s work^24^. The completeness of zinc dissociation in 0.1 N HCl was controlled spectrophotometrically by changing the position of the Soret band maximum. According to the absorption spectra of all the extracts (Fig. 2), the total fraction of Zn porphyrins may reach 82% for dormant cells and 72% for active cells with ALA of the total amount of porphyrins (Fig. 1).

**Figure 3:**
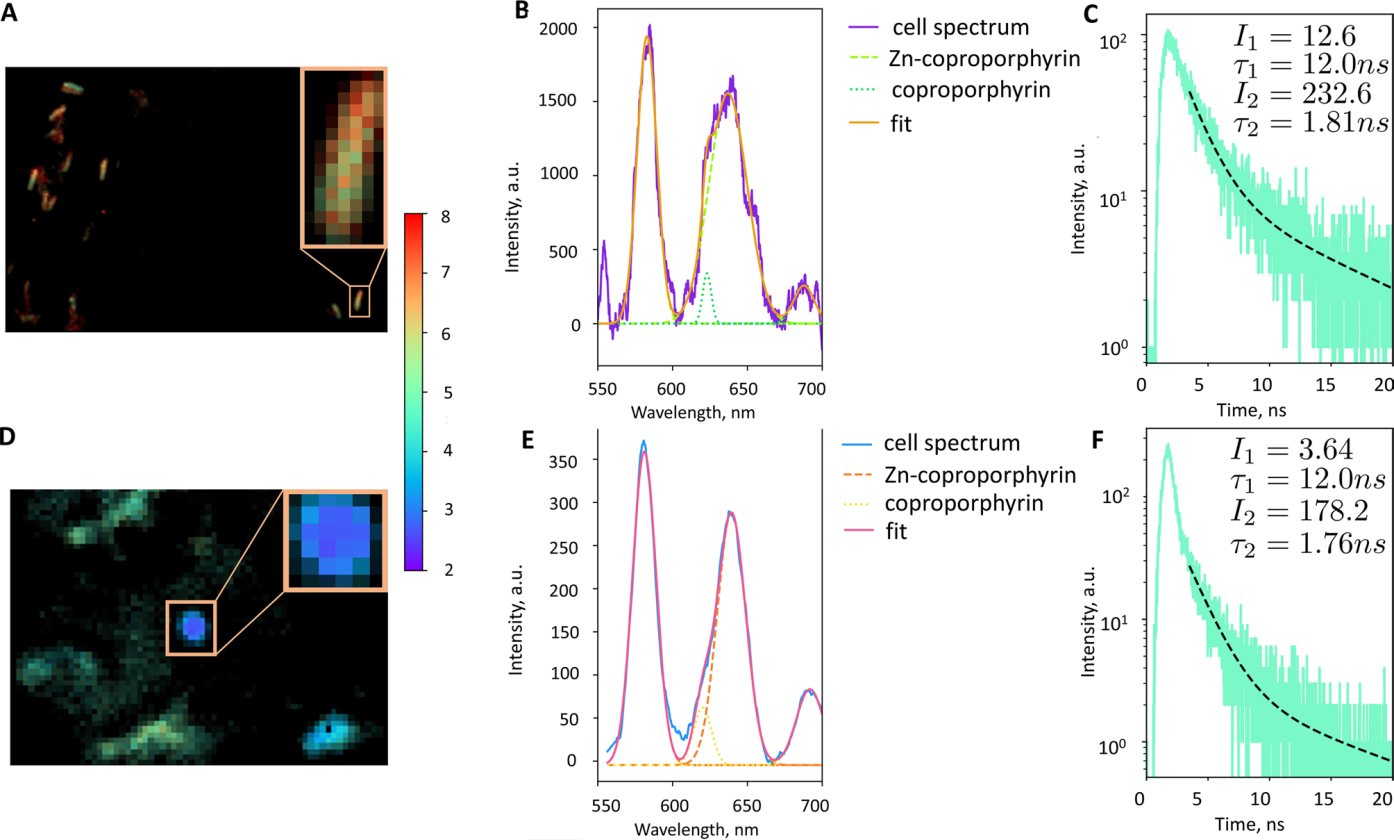
Single mycobacterial cell fluorescence emission spectra for both vegetative and dormant state. 20-day-old vegetative cells and 60-day-old dormant cells were applied to a glass cover slip and left to dry for a few minutes. Remaining cells were fixated with Merckoglass (Merck). Life-time images and fluorescence spectra of single bacteria were recorded by 405 nm laser excitation with 550 nm long-pass emission filter in confocal mode of measurements, (**A**)—life-time images for vegetative cells and (**D**)—for dormant cells with a magnified region of interest at the inserts, (**B**)—fluorescence spectra of porphyrins from single vegetative and (**E**)—dormant bacteria. Life-time decays for vegetative (**C**) and dormant (**F**) cells fit well by two exponential decay with Zn–porphyrin life-time (1.81 ns and 1.76 ns) and free base porphyrin (12.0 ns) correspondetly. Experiments were repeated five times for both vegetative and dormant cells.

An increase in the concentration of porphyrins for single-cell, both vegetative and dormant *Mtb* in the presence of ALA was confirmed by fluorescent confocal microscopy (Fig. 3).

The fluorescence spectrum of single vegetative (Fig. 3b) or dormant (Fig. 3e) live cells of *Mtb* corresponds to the superposition of a large fraction of Zn–porphyrins’ fluorescence and a small fraction of free porphyrins’ fluorescence, similar to that described above (Fig. 2).

The fluorescence lifetimes in a single mycobacterial cell (Fig. 3c,f) are well described by a two-exponential decay with characteristic fluorescence life-times for Zn–porphyrin 1.81 ns for vegetative (Fig. 3c) versus 1.76 ns for dormant (Fig. 3f) and 12.0 ns for both samples for free coproporphyrin that well correlate with the life-time 1.9 ns for Zn–coproporphyrin versus 14.81 ns for coproporphyrin in Triton X-100 (Fig. S5c). Thus, single-cell measurements confirm intracellular localization of porphyrins and Zn–porphyrin in *Mtb* cells for both dormant and vegetative states. Gaussian deconvolution of experimental curves on free base coprpporphyrin and Zn–coproporphyrin fluorescence fit well by these two compounds (strokes and dot curves) and do not show any additional bands from any unknown compounds (Fig. 3b,e).

Flow cytometry analysis showed accumulation of the cell population with red fluorescence during transition to dormancy (Fig. S6). The percentage of fluorescent cells was 7% and 93% for 1 month’s storage and 18 months’ storage, respectively. In the presence of ALA, the percentage of fluorescent cells that contained porphyrins was substantially higher, reaching almost 100% for 18-month-old cells (Fig. S6).

**Figure 4:**
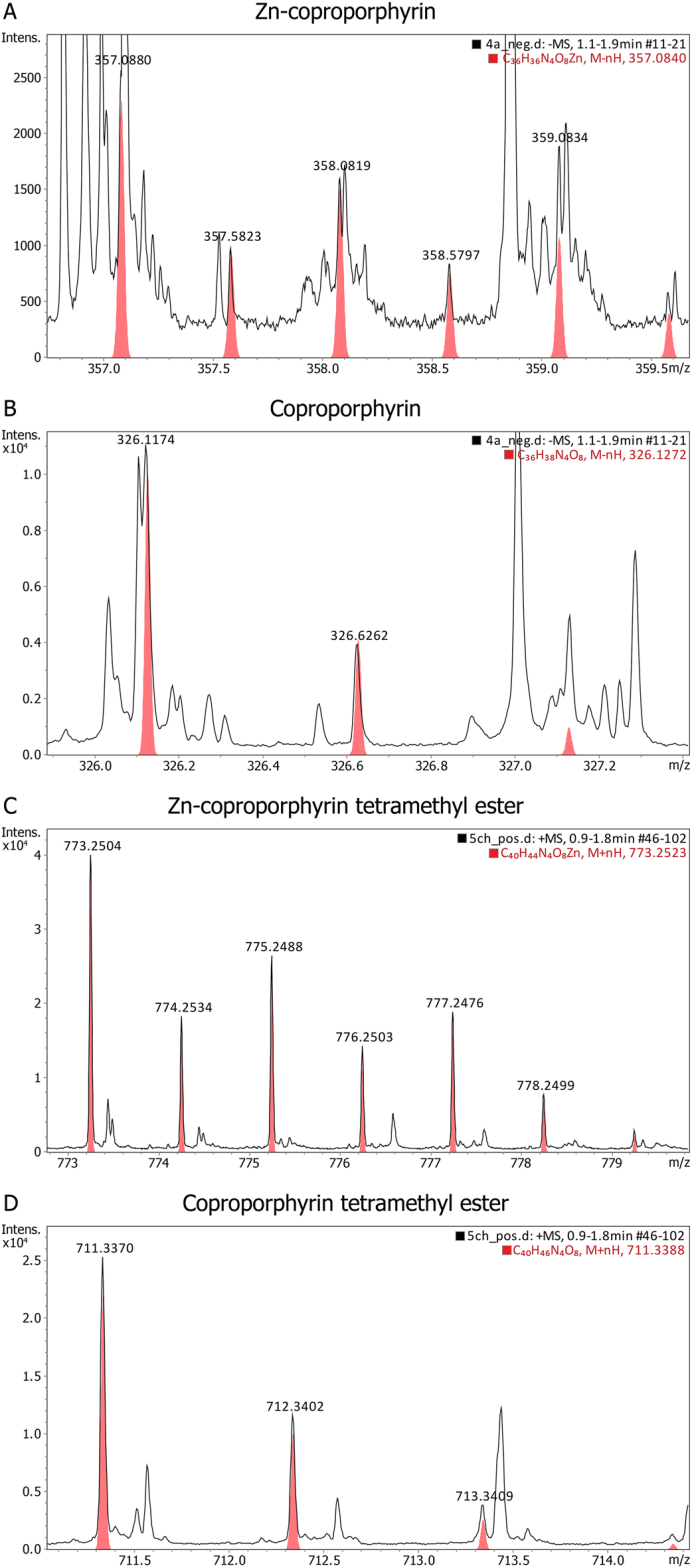
HR-MS spectra in the negative ion mode of extracts of vegetative *M. tuberculosis* cells (**A**, **B**) and in the positive ion mode of extracts of dormant mycobacteria (**C**, **D**) in 75% acetone:15% methanol:10% water solution showing a characteristic isotopologue distribution of Zn–porphyrin complexes (**A**, **C**) and free base coproporphyrin (**B**, **D**), respective calculated distributions overlaid in red. Experiments were repeated three times with similar results.

The obtained chloroform–methanol extracts of porphyrins were analyzed by LC–MS. In the dormant cells obtained in the presence of ALA, the concentration of uroporphyrin, coproporphyrin, and especially coproporphyrin tetramethyl ester increases in dormant *Mtb* cells (Table S1). In vegetative mycobacteria, ALA supplementation primarily stimulates uroporphyrin synthesis and a certain amount of coproporphyrin tetramethyl ester (Table S1). HR-MS analysis made it possible to reveal zinc-containing structures of porphyrins (Fig. 4, Table S2).

Typical Zn isotope pattern^25^ was found for Zn–porphyrins both for active and dormant *Mtb* cells. It was found that, unlike active *Mtb* cells, the unusual derivate of Zn–coproporphyrin, Zn–coproporphyrin tetramethyl ester is present in dormant forms (Fig. 4).

### Transcriptomic analysis of cells of *M. tuberculosis* in a vegetative state and under transition into dormant state upon administration of exogenous ALA

In order to characterize possible biochemical changes in cells caused by ALA addition, a comparative transcriptomic analysis of active and dormant (early stage of transition to dormancy) *Mtb* cultures grown with and without the addition of ALA was carried out. Comparative analysis of sequencing data was carried out in three groups: 1. active mycobacteria vs. dormant ones; 2. active mycobacteria vs. active mycobacteria to which ALA has been added; 3. dormant mycobacteria vs. dormant mycobacteria supplemented with ALA. Changes in expression of 3929 of *M. tuberculosis* genes were analyzed.

**Figure 5:**
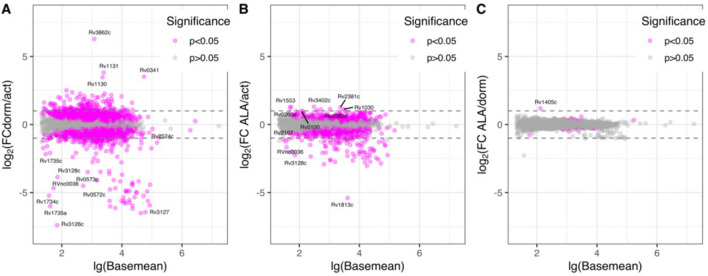
Dependence of the change in the ratio of the level of gene expression in the comparison groups on the normalized average level of *M. tuberculosis* gene expression. The statistical significance of the changes (p value) was calculated based on the Wald statistic. Since multiple comparisons of experimental groups were used, the final value of p was calculated taking into account the Benjamini–Hochberg correction. (**A**) dormant cells versus active cells; (**B**) active cells treated with ALA versus active cells without ALA; (**C**) dormant cells treated with ALA versus dormant cells without ALA.

A graphical analysis of the change in the ratio of the level of gene expression in comparison groups showed in Fig. 5. The maximum differences are found to be dependent on the level of metabolic activity (transition to dormancy ~ metabolically active). Dormant cells show minimum response to ALA supplementation, as shown in Fig. 5.

The transition into the dormant state led to an increase in the expression level of 153 genes (p < 0.05, FC > 2) and a decrease in the expression level of 157 genes calculated based on the comparison of normalized counts. The addition of ALA to actively growing cells resulted in a statistically significant increase in the expression level of 9 genes and a decrease in the expression level of 81 genes based on a comparison of their transcript absolute quantities (Table S3). Similar addition of ALA to dormant cells detects only one gene with statistically significant increased expression: the SAM-dependent methyltransferase *Rv1405c* (which was down-regulated upon transition into the dormant state), the function of which is not annotated (Table S3).

No genes involved in porphyrins metabolism were significantly (FC ˃ 2) impacted in any comparisons. However, as we stated elsewhere, transition into dormancy resulted in a substantial global decrease in mRNA content in *Mtb* cells^26^, which makes it problematic to compare gene expression level in terms of their absolute quantities^27^. Therefore, we also performed differential expression analysis using a standard Z-statistical approach. The Z-standardization procedure reveals some enzymes of porphyrin metabolism plotted on the KEGG map, the expression level of which increases during the transition into the dormant state, as shown in Fig. S7.

Thus, during the transition of *Mtb* cells into the dormant state, activation of genes associated with the metabolism of tetrapyrroles using Z-statistical approach is observed, which can ultimately lead to the accumulation of free fluorescent tetrapyrrole compounds (Fig. S7). Among these genes, the most important are the genes that are located at the beginning of the heme synthesis pathway (*HemB, HemC, HemD*). There is an increase in the expression level of magnesium chelatase (*Rv0958*), which is involved in the formation of magnesium porphyrin, previously identified by mass spectrometry in the lysate of whole *M. tuberculosis* cells^28^, as well as genes responsible for the synthesis of cobyrinates*: Rv2848c* (cobyrinate A, C-diamid synthase, a, c-diamide synthase of hydrobyrinic acid), responsible for the amidation of carboxyl groups in positions A and C of cobyrinate/hydrobrinate^29^*; Rv1314c* (cob(I)irinate a, adenosyltransferase c-diamide), the product of which is a conserved protein found in triton extracts and identified by LC–MS, the function of which remains unknown^30^; *Rv2207* (nicotinate nucleotide dimethylbenzimidazole phosphoribosyltransferase)^30^; *Rv2208* (adenosylcobinamide-GDP ribazole transferase), which is involved in the synthesis of cobalamin 5ʹ-phosphate^29^.

Visualization of the expression level, transformed on their Z-values, revealed ALA-dependent up-regulation of the following genes responsible for the metabolism of porphyrins for actively growing cells: uroporphyrinogen-III synthase (*Rv0260c*) and uroporphyrin-III C-methyltransferase (*Rv0511*) (Fig. S8)—SAM-dependent, membrane-bound methyltransferase methylating uroporphyrin III to form sirohydrochlorin^31^.

The effect of ALA supplementation was also analyzed in relation to the gene expression level of a dormant culture of *Mtb*. The assessment of the Z-normalized gene expression level detects an increase in the expression level of a number of genes of porphyrin metabolism (Fig. S9). In particular, there is an increase in the expression level of the genes of the initial steps of heme biosynthesis: *Rv0512* (porphobilinogen synthase), *Rv0510* (porphobilinogen deaminase/hydroxymethylbilane synthase), and *Rv2677c* (coproporphyrinogen III oxidase). This may contribute to the accumulation of coproporphyrin III in mycobacterial cells.

Thus, transcriptome analysis revealed a certain increase in the activity of a number of genes (albeit different) upon cell transition to dormancy and growth in the presence of ALA associated with the pathways of synthesis and metabolism of porphyrins and precorrins.

### Photodynamic inactivation of *Mtb* cells

In order to elucidate how an increase in intracellular porphyrins concentration caused by the exogeneous addition of ALA influenced aPDI, active and dormant *Mtb* cells grown in the presence of ALA were exposed to LED light with a wavelength of 565 nm (power density 180 mW/cm^2^), for 5–60 min.

**Figure 6:**
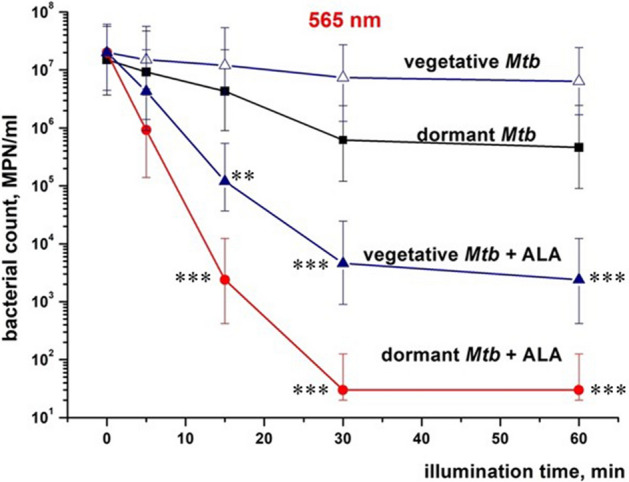
Photodynamic inactivation *M. tuberculosis* cells by light illumination at 565 nm. Dormant and vegetative *Mtb* cells were subjected to PDI as described in “Materials and methods”, at a different time of illumination at 565/24 nm (corresponding to the orange area in Fig. 4), under static conditions. After exposure, cell viability was estimated by MPN assay. Open triangles—vegetative *Mtb*, closed squares—dormant *Mtb,* closed triangles—vegetative *Mtb* grown in the presence of 3 mM ALA. Dormant mycobacteria had zero CFU. The experiments were repeated five times, and a representative result is shown. The MPN method was performed for two biological replicates. Three series of dilutions were made within each replic. Bars demonstrate (95%) confidence limits. Asterisks indicate that the results are significantly different from the control without ALA for every cell group by Student’s t-test. In the case of vegetative mycobacteria, data on the assessment of viable cells assessed by the CFU and MPN methods were close (Fig. S3B), in contrast to dormant forms. For unification, in all cases we used MPN assay. MPN method was performed for two biological replicates in every experiment.

As shown in Fig. 6, illumination of suspension of active cells resulted in a slight decrease in viable cell concentration measured by MPN assay, while the same aPDI for dormant mycobacteria showed a more pronounced effect. Cells grown in the presence of ALA demonstrated very high sensitivity to illumination. In particular, dormant cells were sensitive, with 30 min of illumination resulting in > 99.99% of bacterial killing.

**Figure 7:**
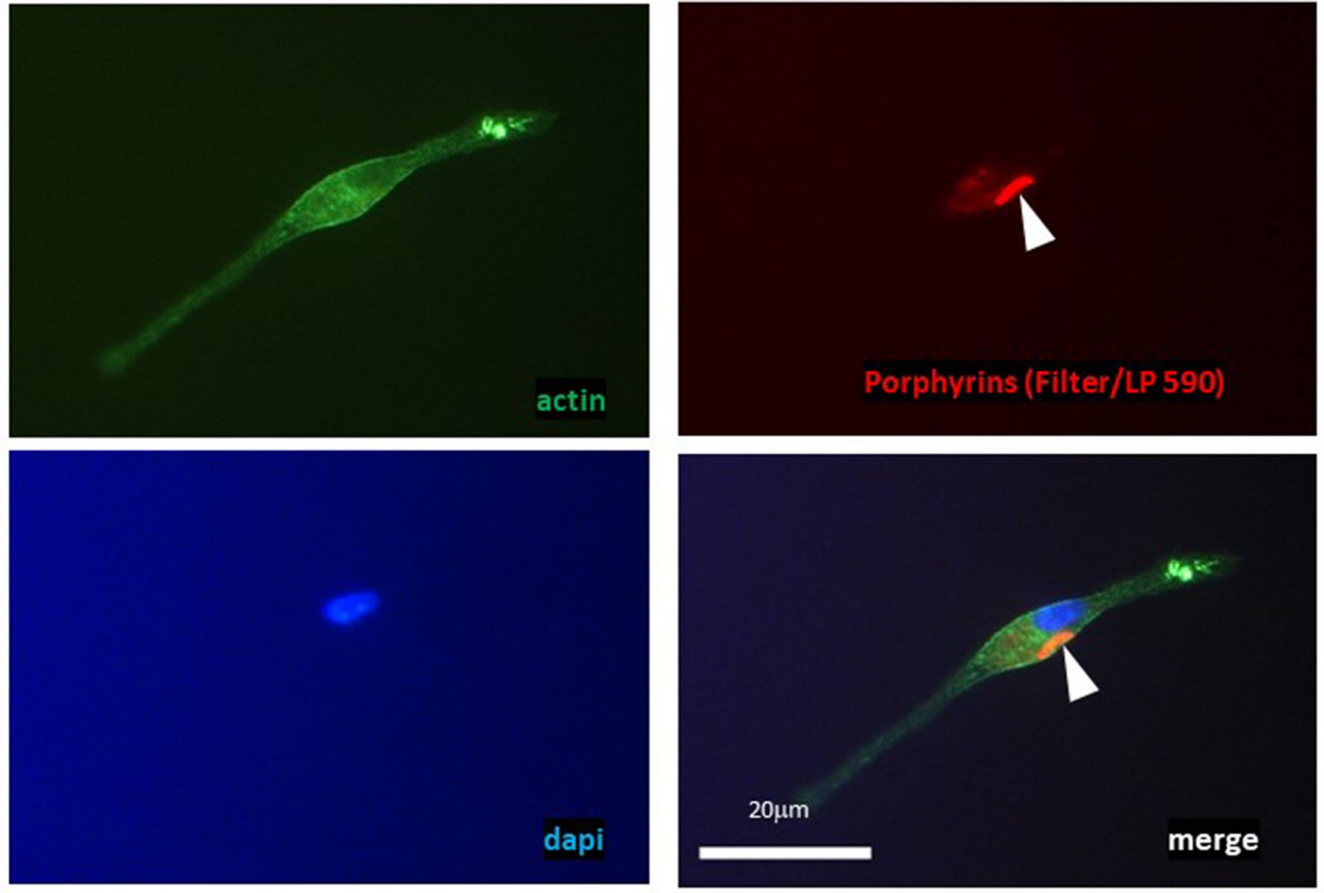
Macrophage containing porphyrin-rich vegetative *M. tuberculosis* cell grown in presence of ALA. Vegetative *Mtb* cells can produce and accumulate porphyrins within 10 days of co-culture with lung macrophages in the presence of 1 mM ALA. In mycobacteria captured by macrophages, fluorescence characteristic of porphyrins was observed. Visualization of red-fluorescent mycobacteria (filter Lp590) within lung macrophage, counerstained with ActinGreen and DAPI (blue). The white arrow points to the mycobacteria inside the macrophage. Experiment was repeated two times with similar results.

**Figure 8:**
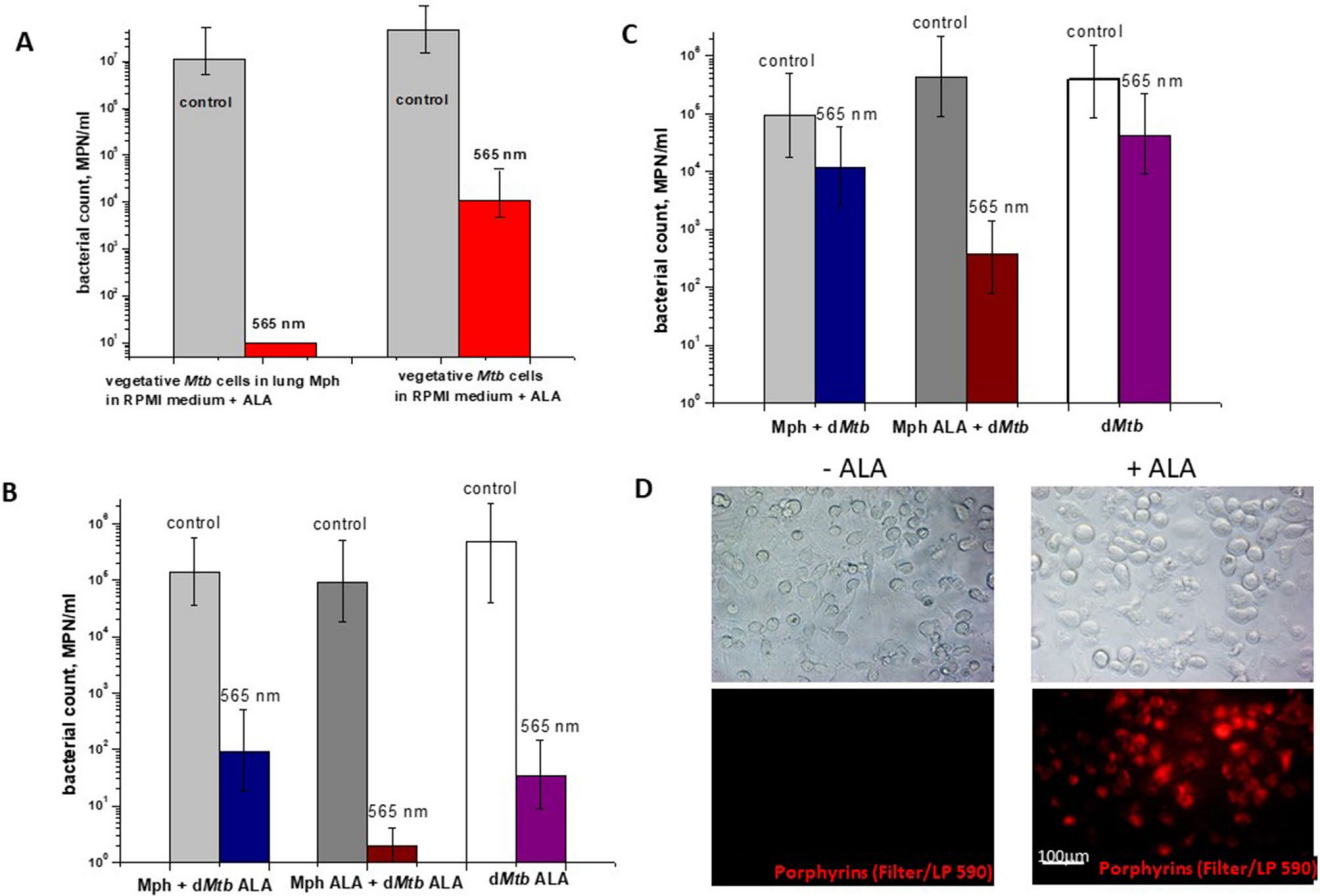
Photodynamic inactivation of vegetative *M. tuberculosis* cells (**A**) and dormant *M. tuberculosis* cells (**B**, **C**) captured by lung macrophages. (**A**) After 10 days of co-culture vegetative mycobacteria with lung macrophages in the presence of 3 mM ALA. (**B**) Macrophages and dormant mycobacteria were grown separately. Dormant mycobacteria were obtained in the presence of 3 mM ALA. Macrophages were obtained in the presence and absence of 3 mM ALA. The bacteria were then captured by macrophages as described in the “Materials and methods”. (**C**) Macrophages and dormant mycobacteria were grown separately. Dormant mycobacteria were obtained in the absence of ALA. Macrophages were obtained in the presence and absence of 1 mM ALA. The bacteria were then captured by macrophages as described in the “Materials and methods”. (**D**) Fluorescence microscopy of macrophages grown in the presence and absence of ALA. Images were performed by invert fluorescent microscope Axio Observer.A1 (Zeiss, Germany), AxioCam MRc5, filter for porphyrins: BP 546/12—FT 580—LP 590. Photoinactivation was performed at 565 nm (300 J/cm^2^). Dormant mycobacteria and vegetative cells displayed zero CFU and 10^7^ CFU before illumination accordingly. Viability of the mycobacteria was estimated by MPN assay. MPN assessment was performed for two biological replicates in every experiment. Bars represent (95%) confidence limits. Experiments were repeated two times with similar results.

Next experiments were performed for *Mtb* cells captured by macrophages, which is closer to the in vivo situation. Previously, mycobacteria transition into dormancy under stressful conditions, including engulfment by lung macrophages during infection has been reported^32^. We assessed in vitro whether or not intracellular *Mtb* produce and accumulate porphyrins during 10-day persistence within purified lung macrophages and in the presence of ALA. As shown in Fig. 7, these culture conditions led to the accumulation of porphyrins by captured bacteria. Remarkably, light sensitivity of mycobacteria captured by macrophages was significantly higher (almost complete sterilization) compared to that of bacilli multiplying in macrophage-free RPMI medium with ALA or conventional growth medium (Fig. 8A).

This difference was possibly due to an increased accumulation of porphyrins inside macrophages. To address this issue, we first incubated macrophages with ALA for 10 days and then added dormant mycobacteria for 16 h before illumination. Visualization of macrophages incubated alone with ALA revealed the appearance of substances with fluorescence corresponding to that of porphyrins (Fig. 8D). Moreover, dormant mycobacteria obtained in the presence of ALA were completely eliminated by light exposure if macrophages were pre-incubated with ALA (Fig. 8B,C). Control dormant cells captured by such macrophages were also sensitive to PDI but to a lesser extent. These results suggest a synergistic effect of macrophages and ALA.

### Influence of photodynamic inactivation on DCPIP reduction by *Mtb* cells

Because a significant amount of accumulated porphyrins in *Mtb* cells grown in the presence of ALA can be extracted with organic solvents/detergents, it is likely that these porphyrins are localized in cell membranes, as was found for *M.smegmatis*^15^. One of the significant membrane-linked processes is electron transport via respiratory chain. We measured DCPIP (dichlorophenolindophenol) reduction at the whole cell level. DCPIP, which is known as an acceptor of electrons, evidently interacted with a pool of reduced menaquinone^33^. Inactivation of menaquinone in isolated bacterial membranes of *M. luteus* results in the inhibition of respiratory chain activity^34^. Illumination of viable *Mtb* cells with an LED 565 nm produced no effect on DCPIP reduction rate. At the same time, cells grown in the presence of ALA showed a decrease in the DCPIP reduction rate up to zero after 15 min of illumination (Table 1). Due to negligible DCPIP reductase activity in dormant *Mtb* cells, such an experiment could not be performed for those cells.

**Table 1:** DCPIP reductase activity of *Mtb* cells.

FDI time (min)	Control (no ALA)	+ ALA
0	0.58 ± 0.01	0.16 ± 0.005
5	0.62 ± 0.01	0.02 ± 0.01
15	0.7 ± 0.05	0
30	0.54 ± 0.01	0
60	0.6 ± 0.02	0

Numbers in table shows change in DCPIP absorbance (OD, 600 nm) during first 20 min after start of the reaction**.** Averaged results of two independent biological replicates, each in three technical replicates, are shown.

## Discussion

In the present study, we found elevated accumulation of porphyrins in dormant *Mtb* (Fig. 1); however, their concentration was low and evidently not enough for successful aPDI (Fig. 6). At the same time, adding ALA to growing *Mtb* before the transition to dormancy significantly increased the accumulation of porphyrins (Fig. 1).

Dormant cells of *Mtb* accumulated a significant amount of porphyrins evidently indicating their possible membrane localization, as was established for *M.smegmatis*^15^. Such localization is likely due to the accumulation of unusual derivates of coproporphyrin–coproporphyrin tetramethyl ester according to LC–MS and HR-MS (Tables S1, S2; Fig. 4) along with copro- and uroporphyrins in cells grown with ALA. The genome of *Mtb* contains several genes coding for possible methyltransferases, which could be responsible for the identified modification of porphyrins.

The luminescence of Zn–porphyrin in microorganisms and mammalian cells were described for many times^35–43^ whilst, the biochemical role of Zn–porphyrins is still not fully understood^43^. In *Mtb*, coproheme (Fe–coproporphyrin III) can be converted to the protoheme by hydrogen peroxide-dependent heme synthase^44,45^. Since Zn–coproporphyrin III cannot bind hydrogen peroxide, it becomes a competitive inhibitor and completely blocks protoheme biosynthesis in this pathway. Such a reversible way of blocking protoheme biosynthesis is in good agreement with the concept of the dormant state.

In our transcriptomic studies, we found that some genes coded for enzymes involved in tetrapyrroles metabolism appeared up-regulated under transition to the dormancy, among which there are the important genes *Rv0512*—(delta-aminolaevulinic acid dehydratase), *Rv0510* (porphobilinogen deaminase), and *Rv0260c* (uroporphyrinogen-III synthase) (Fig. S7). The accumulation of porphyrins may be due to an insufficient amount of ferrochelatase for the conversion of the coproporphyrins to the coproheme for the over-expressed amount of coproporphyrin. The up-regulation of genes involved in the porphyrin biosynthesis pathway and transport was also observed under oxygen and carbon limitation for *M.smegmatis*^46^. At the same time, it was reported that the amounts of four proteins involved in porphyrin biosynthesis were increased in *Mtb* cells under starvation conditions (HemC, CysG, HemZ, and Rv1314c)^47^*.* Perhaps the different response of enzymes involved in porphyrin biosynthesis in our study and in published results could be explained by a substantial difference in establishing non-replicative conditions in both cases.

At the same time, the presence of ALA in growth medium leads to the activation of two genes in the initial part of the porphyrin biosynthesis pathway (Figs. S8, S9); however, the activity of two genes responsible for terminal conversion of coproporphyrin either did not change or down-regulated (hemY) protoporphyrin IX magnesium-chelatase (EC 6.6.1.1.) (Figs. S8, S9). In this case, the incorporation of zinc into the porphyrin macrocycle can proceed non-enzymatically at a fairly high rate^48^.

A block for further consumption of porphyrins could also be established due to a decrease in protein biosynthesis at dormancy and a lack of final protein acceptors of protoheme.

The addition of ALA to bacterial culture dramatically increases the sensitivity of viable *Mtb* cells to illumination at 565 nm, where both coproporphyrin and Zn–coproporphyrin absorb light (Fig. 2). The most significant factor in this case may be illumination at the Zn–porphyrin absorption wavelength. Zn–porphyrin has a long-lived triplet state, which is known to play a decisive role in photodynamic therapy of tumors^49^. This effect of ALA was preserved for vegetative and dormant bacteria captured by lung macrophages and even enhanced if macrophages were precultivated with ALA, revealing a synergetic effect (Fig. 8). Whilst the experiments performed in this study are in favor of the causality of ALA→porphyrins→photosensitivity, the genetic proof of it in future would be very valuable.

The application of aPDI for inactivation of pathogenic bacteria has acquired significant attention in recent years. In this regard, a variety of exogenous photosensitizers are most common in killing bacteria^50^ including mycobacteria^4^, among which substances of a porphyrin nature were applied. Thus, *Mtb* with multiple antibiotic resistance was inactivated by aPDI using exogenous radachlorin^51^. Exogenously added porphyrins as photosensitizers were successfully applied for inactivating *Mycobacterium fortutinum*^52^, *M. bovis* BCG^53^, *M. marinum*^54^*,* and *M.smegmatis*^55^ in vitro and in vivo.

However, due to the known limitations of exogenous photosensitizers for aPDI in vivo (problems of specificity, delivery to bacteria, penetration through bacterial cell wall) prompted the use of endogenous photosensitizers like those ubiquitously present in living cells—porphyrins of different structures. Induction of porphyrin synthesis in cells by ALA is the most well- known approach in PDI for anticancer therapy^56,57^. Recently, this approach has been used to inactivate pathogenic bacteria such as *Str. oralis* on the surface of titanium implants^58^, *Mycobacterium abscessus*, leading to skin infections, in vitro^59,60^*.* Successful application of ALA-PDI was demonstrated for the curing of non-tuberculosis infection and cancer in clinic^61–64^. However, in the above experiments, ALA-PDI resulted in a decrease in bacterial viability for fast-growing vegetative bacteria measured by CFU by not more than one order of magnitude (for example, for *Mycobacterium abscessus* grown in presence of ALA, CFU changed from 7E7 to 4E7 after illumination^60^), which is far from full sterilization. Highly effective ALA-PDI found in the present study for active and especially dormant *Mtb* (Fig. 6) could reflect peculiarities of metabolism of slow-growing mycobacteria in active and non-replicative states. In the last case, absence of polymer biosynthesis de novo^18^ should preclude resynthesis of damaged enzymes, including those involved in defense against radicals and active forms of oxygen. Evidently, the harmful conditions developed inside of macrophages after bacterium capturing in addition to ALA-mediated accumulation of host porphyrins enhance sensitivity of bacterial cells to aPDI, as was found experimentally (Fig. 8). Almost full sterilization of *Mtb* cells captured by macrophages makes this approach potentially useful for further medical application as alveolar macrophages are the first-line responders against *Mtb*.

However, we should admit that physiological state of the pathogen in macrophages, in replicating state in vitro and in in vitro dormancy model may not be identical. Indeed, dormant forms in vitro are much more sensitive to light than active forms, both grown in the presence of ALA (Fig. 6). However, for both the active forms (Fig. 8a) and the dormant forms (Fig. 8c) the PDI effect is comparable and almost complete sterilization is achieved. Therefore, mechanisms responsible for PDI killing of mycobacteria within macrophages may differ from it found in vitro. Evidently, more efforts are needed to extend the found PDI effects in this study to in vivo situation using laboratory animals.

The possible targets of antibacterial PDI in bacterial cells are the subject of continuing discussion, although primary destructive generation of reactive oxygen species upon PDI is a commonly accepted process^50^. Among putative targets, lipids via their peroxidation, nucleic acids, and proteins are under consideration^50^. The negative effect of PDI on iron metabolism and development of ferroptosis has been demonstrated for *Mycobacterium abscessus*^59^*.* In this study, we found a prominent effect of ALA-PDI on the functioning of endogenous respiration of mycobacteria (Table 1). This effect could be caused by either a decrease in respiratory substrate flow or direct inhibition of the respiratory chain. The last suggestion is plausible because of membrane localization of significant amount of porphyrins induced by ALA. *Mtb* respiratory contains succinate dehydrogenase with a membrane-bound FeS cluster^65^, which is known as a sensitive target for ROS^66^ and therefore for PDI in presence of endogenous porphyrins. As suggested, succinate dehydrogenase (or structurally close fumarate reductase) may play an important role in maintaining of energized membrane in hypoxic non-replicating *Mtb* cells^67^. In addition, indirectly, PDI inducing modification of membrane lipid matrix^68^ could influence respiratory chain activity. Evidently, to make a final conclusion on the involvement of respiratory chain in the identified effect an additional study with isolated mycobacterial membranes is needed. Future experiments should also clarify how found inhibition of cell respiration after illumination impacts on the drop in cell viability.

Thus, for the first time, we demonstrated the successful application of aPDI for the inactivation of *Mtb* cells in vitro and in macrophages associated with accumulation of endogenous porphyrins in the presence of 5-aminolevulinic acid.

In conclusion, the findings of this study provide a new perspective for the eradication of vegetative, including multidrug-resistant, and dormant forms of *Mtb* using endogenous porphyrins as photosensitizers. It is logical to apply this approach first for TB with surface localization. However, using current progress in clinical development of photodynamic therapy of cancer^64^, especially different light illumination techniques, including interstitial PDT, and endoscopic technic, localized TB focuses including those in the lungs and other organs could also be tackled by PDI.

## Materials and methods

### Formation of the dormant forms of *M. tuberculosis* upon the medium self-acidification

Inoculum was initially grown from frozen stock stored at − 70 °C in 40% glycerol. To obtain dormant forms, we used the model of environmental acidification^18,69^. *Mtb* strain H37Rv was grown for 8 days (up to OD600 = 2.0) in Middlebrook 7H9 liquid medium (Himedia, India) supplemented with 0.05% Tween 80 and 10% growth supplement ADC (albumin, dextrose, catalase) (Himedia, India). Next, 1 ml of the initial culture was added to 200 ml of modified Sauton medium that contained (per liter) the following: KH_2_PO4, 0.5 g; MgSO_4_⋅7H_2_O, 1.4 g; l-asparagine, 4 g; glycerol, 2 ml; ferric ammonium citrate, 0.05 g; citric acid, 2 g; 1% ZnSO_4_.7H_2_O, 0.1 ml; pH 6.0–6.2 (adjusted with 1 M NaOH) and supplemented with 0.5% BSA (Cohn Analog, Sigma), 0.025% tyloxapol and 5% glucose. Cultures were incubated in 500 ml flasks that contained 200 ml modified Sauton medium at 37 °C with shaking at 200 rpm (Innova, New Branswick, NJ, USA) for 30–50 days, and pH values were periodically measured. In log phase, pH of the culture reached 7.5–8 and then decreased in stationary phase. When the medium in post-stationary phase *Mtb* cultures reached pH 6.0–6.2 (after 30–45 days of incubation for different experiments), cultures (50 ml) were transferred to 50 ml plastic tightened capped tubes and kept under static conditions, without agitation, at room temperature. At the time of transfer 2-(*N*-morpholino) ethanesulfonic acid (MES) was added in a final concentration of 100 mM to dormant cell cultures to prevent fast acidification of the spent medium during long-term storage. To induce the synthesis of porphyrins, 3 mM 5-aminolevulinic acid (ALA) was added to the cultures during the transition of mycobacteria into a dormant state (20 days after inoculation).

For the vegetative state, *Mtb* cells were cultivated in Middlebrook medium (Himedia, India) supplemented by ADC (Himedia, India) and 0.05% of Tween-80 with the addition of 3 mM 5-aminolevulinic acid (5 days after inoculation). Cultures were cultivated for 14 days.

### Extraction of porphyrins

The porphyrins were extracted from the biomass according to Bligh^70^. Bacteria were washed three times with 50 mM phosphate buffer (pH 7.4). Then, 1 ml of chloroform and 2 ml of methanol were added to the wet biomass of the cells (0.8 g). Cells were agitated for 12 h in the extraction mixture with subsequent centrifugation (4000*g*, 20 min). The next extraction was performed during 12 h using 2% Triton X-100 in 20 mM Tris HCl buffer at pH 7.4.

For high-resolution mass spectrometry (HRMS), the porphyrins were extracted from dormant and vegetative mycobacteria 0.8 g the wet biomass of the cells using 3 ml of 75% acetone, 15% methanol with 10% water, or 3 ml of chloroform:methanol:water (1:2:0.8)^70^.

### Spectrophotometric, fluorescence, and phosphorescence analysis of porphyrin in mycobacterial extracts

The absorption spectra of the extracts were recorded in a 1 cm cuvette on a spectrophotometer Cary-50. Excitation and fluorescence spectra were recorded in a 3 mm cuvette on a Cary Eclipse spectrofluorometer in both fluorescence and phosphorescence modes at room temperature. To record the phosphorescence spectra, the extracts were mixed in a ratio of 1/10 with a deoxygenating solution containing sodium bisulfite (40 mg/ml NaHSO_3_, 2% Triton X-100, pH 7.8) as described in^71^. Phosphorescence lifetimes were recorded in the same deoxygenating solution on a Cary Eclipse spectrofluorometer using the Lifetime program.

### LC–MS

Porphyrin identification in chloroform–methanol extracts was carried out by LC–MS on an Agilent 1290 Infinity chromatograph (USA) with an Agilent 6460 TQ mass spectrometer (USA).

An Agilent Eclipse Plus C18 1.8 um 2.1 × 50 mm column was used with mobile phase: A—0.1% formic acid in water, B—0.1% formic acid in acetonitrile; Gradient A–B; column temperature—40 °C, flow rate—0.4 ml/min, detection—UV/VIS 400 nm, MS ESI(+) full scan. The mass spectra were acquired with positive electrospray ionization (ESI) with the ion spray voltage set at 5.5 kV. We used 1 μg of the porphyrin standards for the initial tests. Coproporphyrin III, Uroporphyrin III, Coproporphyrin III Tetramethyl Ester and Zn–coproporphyrin III were kindly provided by Prof. Geliy Ponomarev and used as standards. Due to the use of 0.1% formic acid in this type of LC–MS analysis, separate data on Zn–porphyrins were lost and included in the total amount as free porphyrins.

### High-resolution mass spectrometry (HR-MS)

The presence of Zn–porphyrins was confirmed by HR-MS. HR-MS analysis of extracts was performed on an Impact II QqTOF high-resolution mass-spectrometer (Bruker Daltonik, Germany) equipped with Apollo II ESI ion source (Bruker Daltonik, Germany) under the following conditions: direct sample infusion at 30 µL/min, HV capillary at 4.5 kV for positive mode and 3.0 kV for negative mode, spray gas—nitrogen at 0.9 bar, dry gas—nitrogen at 4.0 L/min 200 °C, scan range m/z 50–1200, 0.2 Hz scan rate, automatic internal calibration with sodium trifluoroacetate solution. Spectra were processed with Compass DataAnalysis 5.1 (Bruker Daltonik, Germany). Zinc is naturally present as 5 isotopes; ^64^Zn: 45.89%, ^66^Zn: 27.81%, ^67^Zn: 4.11%, ^68^Zn: 18.57% and ^70^Zn: 0.62%, and in Fig. 4 the magnification of the range of interest clearly confirms the presence of a Zn–coproporphyrin with the detection of six negative isotopologue ions and Zn- coproporphyrin tetramethyl ester with detection of six positive isotopologue ions in acetone/methanol/water extracts.

### Single cell confocal fluorescence microscopy, life-time measurements, and microspectrofluorimetry

Vegetative cells of *M. tuberculosis* cells were grown in Middlebrook 7H9 liquid medium supplemented by ADC and Tween-80 for 10 days (initial bacteria concentration 10^5^ cells per ml). After 3 days post inoculation 3 mM ALA was added to some flasks. Dormant *Mtb* cells were obtained in modified Sauton’s medium as described in “Materials and methods”. ALA (3 mM) was added after 20 days post inoculation.

Sample preparations: A volume of 2 ml of 10^9^ *Mtb* cells was centrifuged at (8000 rpm for 10 min); the bacterial pellet was washed with water twice and then resuspended in distilled 5 ml of water. After that, the suspension was applied to a glass cover slip and left to dry for a few minutes, then fixated with Merckoglass (Merck). After solidification of the Merckoglass, samples were used for microscopy.

Fluorescence lifetime measurements were performed on a PicoQuant MicroTime 200 confocal scanning system (Pico-Quant GmbH, Berlin, Germany) based on an Olympus IX-71 inverted fluorescence microscope (Japan). A high numerical aperture apochromatic objective (UApo N 100X, NA 1.49, oil immersed, Olympus) was used for excitation at 405 nm by an LDH-P-C-400B laser (Pico-Quant GmbH, Berlin, Germany) and emission was recorded using a 550 nm Longpass filter with an avalanche photodiode (SPAD, Perkin Elmer) using the TCSPC mode with a PicoHarp 300 board. SymphoTime® software was used for data collection. Original data obtained via microscope were recalculated to form FLIM images. In order to extract precise estimates of fluorescence lifetimes, histograms of pixels that belonged to cell areas were summing. The resulting histogram was then fitted with a biexponential decay model. The weighted least-squares approach was applied due to the heteroscedasticity largely presented in the data^72^. The BFGS (Broyden-Fletcher-Goldfarb-Shanno) algorithm was used for optimization because of its better performance in problems with large variation in data^73^.

In order to prove the presence of porphyrins, single cell microspectrofluorimetry was used. Fluorescence spectra were recorded in the confocal mode of the MicroTime 200 system using a Shamrock 163 spectrograph with a Newton DU-970 camera (Andor, UK). Long-pass 550LP filter was used. Spectrographs were calibrated using reflection of the laser beam from a glass cover slip. Background subtraction of the single-cell fluorescence spectra was carried out according to the algorithm^74^. Obtained fluorescence spectra were smoothed with a Savitzky–Golay filter. Data analysis was performed using the MicroCal Origin 8.5 Pro software and Python 3.10.9 with additional packages for data analysis such as Numpy 1.22.2, SciPy 1.8.0, and Symfit 0.5.5. Matplotlib 3.5.1 package was used for data visualization.

For porphyrin visualization within the lung macrophages and engulfed mycobacteria cells were cultivated on coverslips in the presence of mycobacteria, as described below. At indicated time points, cells were fixed with 1% PFA for 10 min, washed with × 1 PBS and stained with ActinGreen-488 (MolecularProbes by LifeTechnologies) and DAPI. Photography was performed under microscope AxioSkop40 (Zeiss, Germany), magnification × 40. Macrophages, incubated alone with ALA, were procured for visualization directly in plate for cultivation. Photography was performed under inverted fluorescent microscope Axio Observer.A1 (Zeiss, Germany), AxioCam MRc5, filter for porphyrins: BP 546/12—FT 580—LP 590. Source data is available on the website 10.5281/zenodo.8322146.

### Flow cytometry analysis and microscopy

The *Mtb* cells were fixed by 2% formaldehyde for 24 h. Aliquots of bacterial samples—500 μl (OD600 ~ 0.2) were placed into the flow-cytometry plastic tubes. The CytoFlexS system (Beckman Coulter, USA) was used to measure porphyrin fluorescence of the bacterial cells.

The graphs were analyzed in coordinates FSC-A vs FL3-A (forward scattering vs. red fluorescence); the voltage on the detectors: FSC-A-10, FL3-A-50. In total, 10,000 events were acquired at an analysis rate of 1000 events. Data analysis was carried out using the CytExpert software for the CytoFlex platform.

Phase-contrast and epifluorescence microscopy was carried out using a Nikon eclipse Ni-U microscope. Fluorescence microscopy was carried out in the FITC channel (EX = 465–495, DM = 505, BA = 515–555). Magnification × 1500. Photos were taken using a Nikon DSQi2 camera (Japan). Analysis of the morphological changes was performed using the NIS-Elements BR software.

### Isolation of lung macrophages

Mice of inbred strain C57BL/6JCit (B6) were bred and maintained under conventional conditions at the Animal Facilities of the Central Tuberculosis Research Institute (CTRI, Moscow, Russia) in accordance with guidelines from the Russian Ministry of Health # 755, and under the NIH Office of Laboratory Animal Welfare (OLAW) Assurance #A5502-11. Water and food were provided ad libitum. Female mice of 10–12 weeks of age were used. All experimental procedures were approved by the CTRI Institutional Animal Care and Use Committee (IACUC protocols #2, 7, 8, 11, approved on March 6, 2021). For adherence of macrophages to plastic, RPMI 1640 supplemented with 10% fetal calf serum (FCS), 10 mM HEPES, 2 mM l-glutamine, 1% nonessential amino acids, 1 mM pyruvate, 5 × 10^–5^ M 2-mercaptoethanol, and antibiotics (all components from HyClone, Carlington, The Netherlands) was used (medium 1). All assays were performed in a medium which differed from medium 1 in that it contained no antibiotics and only 2% FCS (medium 2) or 5% FCS (medium 3).

Lungs were enzymatically digested as described previously^75,76^. Briefly, blood vessels were washed out by heart perfusion with 0.02% EDTA-PBS through the right ventricle and cut vena cava, lungs removed, sliced into 1–2 mm^3^ pieces, and incubated at 37 °C for 90 min in supplemented RPMI-1640 containing 200 U/ml collagenase and 50 U/ml DNase-I (Sigma, MO). Single-cell suspensions were obtained by vigorous pipetting. The lung cells were washed twice in Hanks’ balanced salt solution (HBSS) containing 2% FCS and antibiotics and resuspended in medium 1. Cells (20 × 10^6^ to 30 × 10^6^) were incubated in 10 ml of medium 1 for 1.5 h on 90-mm-diameter treated Petri dishes (Costar-Corning, Badhoevedorp, The Netherlands) at 37 °C, 5% CO_2_. Nonadherent cells were removed by triple vigorous washing with warm antibiotic-free HBSS containing 2% FCS. Adherent cells were detached from the plastic by incubating the monolayers in 0.02% EDTA-PBS antibiotic-free solution for 30 min at room temperature. Cell suspensions were obtained by pipetting, washed twice with HBSS, and resuspended in medium 2.

### Macrophage-mycobacteria co-culture

To obtain monolayers of good density, 5 × 10^4^ lung macrophages were plated in a well of a flat-bottom 96-well plate (Costar-Corning). Macrophages adhered for 1 h before live *M. tuberculosis* were added at the MOIs indicated in the figures and tables. After 14 h incubation, wells were gently washed to remove extracellular bacteria; then, warm medium 3 containing 1 mM ALA was added (200 μl per well). In 5 days of co-culture, 4 × 10^4^ of fresh macrophages were added to phagocyte extracellular bacteria released from dying macrophages. After 11 days of total cultivation, the wells were washed from extracellular bacteria and *Mtb*-containing macrophages underwent illumination.

To test the possible bactericidal activity of porphyrins gained by macrophages during cultivation in the presence of ALA, we cultured lung macrophages in the presence or absence of 1 mM ALA for 10 days and then added 4-month-old dormant *Mtb* obtained in the presence or absence of ALA at MOI = 1. After 16 h of incubation, wells containing *M. tuberculosis* were gently washed to remove extracellular bacteria, and *Mtb*-containing macrophages underwent illumination.

Photos were taken using an AxioSkop40 microscope (Zeiss, Germany) and AX10 filter for porphyrins: BP 546/12—FT 580—LP 590.

### Viability evaluation by MPN

Most probable number (MPN) assays of *Mtb* were performed in 48-well plastic plates (Corning) containing 1 ml of special medium for the most effective reactivation of dormant *Mtb* cells^69^. The medium contained 3.25 g nutrient broth dissolved in 1 L of a mixture of Sauton medium (0.5 g KH2PO4; 1.4 g MgSO_4_.7H_2_O; 4 g l-asparagine; 0.05 g ferric ammonium citrate; 2 g sodium citrate; 0.01% (w/v) ZnSO_4_.7H_2_O per liter pH 7.0), Middlebrook 7H9 liquid medium (Himedia, India), and RPMI (Thermo Fisher Scientific, USA) (1:1:1) supplemented with 0.5% v/v glycerol, 0.05% v/v Tween 80, 10% ADC (Himedia, India). *Mtb* cells were serially tenfold diluted in the reactivation medium. Appropriate five serial dilutions of *Mtb* cells (100 μl) were added to each well containing the same medium in triplicate. Plates were incubated at 37 °C for 21 days. Wells with visible bacterial growth were counted as positive, and MPN values were calculated using standard statistical methods^77^.

### Isolation of RNA, Illumina sequencing

ALA was added to the cultures in a final concentration of 3 mM and the cultures were incubated for the next 2 h. Bacterial samples (active *M. tuberculosis* (MAC_1, MAC_2, MAC_3), dormant (DORM_1,DORM_2,DORM_3), active in the presence of 5-aminolevulinic acid (ALA)(MACALA_1,MACALA_2, MACALA_3), and also dormant in the presence of ALA (DORMALA_1, DORMALA_2, DORMALA_3) were chilled in triplicate on ice, harvested by centrifugation (4000*g*, 15 min, 4 °C), and 1 ml Trizol reagent was added to the pellets. Cells were disrupted using zirconia beads (0.1 mm) in a BeadBeater (Biospec, USA) with 100 μm zirconium beads, and centrifuged (4000*g*, 45 min, 4 °C) to clear cell lysates from cell debris. After centrifugation, the supernatant was extracted once with chloroform. Nucleic acids were then precipitated with isopropanol, harvested by centrifugation, washed with 70% ethanol and re-dissolved in nuclease-free water (Promega, USA). RNA was then isolated using a RNeasy Mini kit (Qiagen). Each RNA sample was finally treated with RNase-free Turbo DNase (Life Technologies, Carlsbad, CA, USA) and purified with the RNeasy mini kit (Qiagen, Venlo, The Netherlands).

After RNA isolation, removal of excess ribosomal RNA as well as cDNA synthesis was performed using the Illumina TruSeq Stranded Total RNA With Illumina Ribo-Zero Plus rRNA Depletion kit (Illumina, San Diego, CA, USA). The resulting cDNA was used to prepare libraries compatible with Illumina sequencing technology.

### Processing of RNA-seq data

The quality of the resulting libraries was checked using the Fragment Analyzer. Quantitative analysis was performed by qPCR. After quality control and evaluation of the DNA quantity, the pool of libraries was sequenced on an Illumina NovaSeq 6000 instrument (length of reads—150 bp on both sides of the fragments). FASTQ files were generated using bcl2fastq Conversion Software v2.20 (Illumina, San Diego, CA, USA). As a result, 1,107,614,502 reads were received. The quality control of the sequencing results was carried out using the FastQC program (https://www.bioinformatics.babraham.ac.uk/projects/fastqc/). Reads were filtered and adapter sequences were removed using TrimGalore and Cutadapt (https://github.com/FelixKrueger/TrimGalore).

### Sequencing analysis and mapping to *M. tuberculosis* H37Rv reference genome

Four variants of cultures were used for the analysis in three biological replicates each: (1) actively growing vegetative mycobacteria—10 days of growth on a standard Sauton medium; (2) actively growing mycobacteria incubated for 2 h with 3 mM ALA; (3) mycobacteria at an early stage of transition into a dormant state—30 days; (4) mycobacteria at an early stage of transition to a dormant state incubated for 2 h with 3 mM ALA.

After quality control evaluation and pruning of low-quality reads, the reads were mapped to the *M. tuberculosis* reference genome (https://www.ncbi.nlm.nih.gov/nuccore/NC_018143.2). Reads were mapped to the reference genome using HiSat2^78^. The quality of the mapping results was assessed using RSeQC^79^. As a result of filtering out unsatisfactory reads, the percentage of reads mapped to the reference *M. tuberculosis* genome was ~ 97% of the total number of mapped reads. The expression level was calculated using HTSeq^80^, and the subsequent bioinformatic analysis of ready-made expression matrices was carried out using DESeq2^81^.

A table containing the data of the expression levels of all genes was added as Supplementary Materials Table S0. The Z-score was calculated from the mean and standard deviation of the reference dataset^82^.

### Photodynamic inactivation

Suspensions of dormant/active *Mtb* with OD = 0.1, which corresponds to ca. 10^7^ bacteria per ml, were used for light inactivation experiments. Volumes of 100 μl were pipetted into the wells of a 96-well plate (Nunc). Illuminations of the samples were performed by LED SOLIS-4C (Thorlabs, USA) and interference filters with the band passes 565/24 nm (MF565-24, ThorLabs, USA) that correspond to the absorption peaks for both coproporphyrin and Zn–coproporphyrin (Fig. 4). Light power was measured by a Newport 2936-c power meter and was equal to 180 mW. Light beam was collimated to the diameter 6.5 mm that corresponded to the diameter of wells of a 96-well plate. Illumination coupling MT-CON (Thorlabs, USA) with optimized optics for bright bottom homogenous illumination of suspension in the wells was used. Illumination was for 5-, 15-, 30-, or 60-min. Temperature control was performed by Fluke 80BK Type K Multimeter Thermocouple Temperature Probe directly in the microwell before and after illumination and in the presence of and without bacterial suspension with the precision ± 0.2 °C.

Temperature was below 40 °C in the wells during all the experiments.

After illumination of the samples, serial tenfold dilutions (10^−1^ to 10^−7^) were prepared and bacterial viability was evaluated by MPN assay.

### DCPIP reduction

Endogenous respiration was determined by reduction in DCPIP (2,6-dichlorophenolindophenol) in the presence of menadione monitored spectrophotometrically at 600 nm. The reaction mixture (4 ml) contained 0.2 μmol 2,6-DCPIP, 0.6 μmol menadione, and 400 μl of the cell suspension in Sauton medium (pH 7.4).

### Statistical analysis

Statistical processing was carried out via the analysis of the standard deviation or relative error within the data group. All the data are presented as the mean values of three replicate determinations. MPN values were determined using de Man’s tables calculated on the basis of a Poisson distribution^77^. For the MPN assay, (95%) confidence limits were calculated. The MPN values were considered statistically different if low and high confidence limits were not overlapping. Student’s test assuming unequal variance was performed for estimation of significance for comparative data. P-values are indicated as follows: **p < 0.01, ***p < 0.001.

The essentiality of statistics was determined at p < 0.05. The OriginPro software package 8.5 and Design Expert Software Version 8.0.6 were involved in the statistical analysis.

The corresponding p-values of comparison of the three groups of cells (dormant vs. active, dormant vs. dormant + ALA, active vs. active + ALA) in transcriptomic analysis were calculated from a two-sided t-test.

### Ethical statement

Authors confirming the study is reported in accordance with ARRIVE guidelines (https://arriveguidelines.org). All experimental procedures were approved by the CTRI Institutional Animal Care and Use Committee (IACUC protocols #2, 7, 8, 11, approved on March 6, 2021).

### Supplementary Information

Supplementary Table S0.

Supplementary Information.

## Data Availability

The datasets generated and/or analysed during the current study are available in the ZENODO repository 10.5281/zenodo.8322146) or GEO SRA accession SRR26142264 (MAC_1), SRR26142263 (MAC_2), SRR26142260 (MAC_3), SRR26142259 (DORM_1), SRR26142258 (DORM_2), SRR26142257 (DORM_3), SRR26142256 (MACALA_1), SRR26142255 (MACALA_2), SRR26142254 (MACALA_3), SRR26142253 (DORMALA_1), SRR26142262 (DORMALA_2), SRR26142261 (DORMALA_3).
