# Supplementary material for: Acquiring of photosensitivity by Mycobacterium tuberculosis in vitro and inside infected macrophages is associated with accumulation of endogenous Zn–porphyrins
Source: Sci Rep. 2024 Jan 8;14:846. doi: 10.1038/s41598-024-51227-z (PMC10774309; doi:10.1038/s41598-024-51227-z)
Supplement: Supplementary file 2 — Supplementary Information. [file 41598_2024_51227_MOESM2_ESM.docx]

**Supplementary**

**Acquiring of photosensitivity by *Mycobacterium tuberculosis* *in vitro* and inside infected macrophages is associated with accumulation of endogenous Zn-porphyrins**

Margarita O. Shleeva^1^, Irina A. Linge^2^, Ivan A. Gligonov^1*^, Galina N. Vostroknutova^1^, Denis M. Shashin^1^, Andrey M. Tsedilin^1^, Alexander S. Apt^2^, Arseny S. Kaprelyants ^1^ and Alexander P. Savitsky^1^

^1^A.N. Bach Institute of Biochemistry, Federal Research Centre ‘Fundamentals of Biotechnology’ of the Russian Academy of Sciences, Moscow, Russia

^2^Laboratory for Immunogenetics, Central Tuberculosis Research Institute, Moscow, Russia


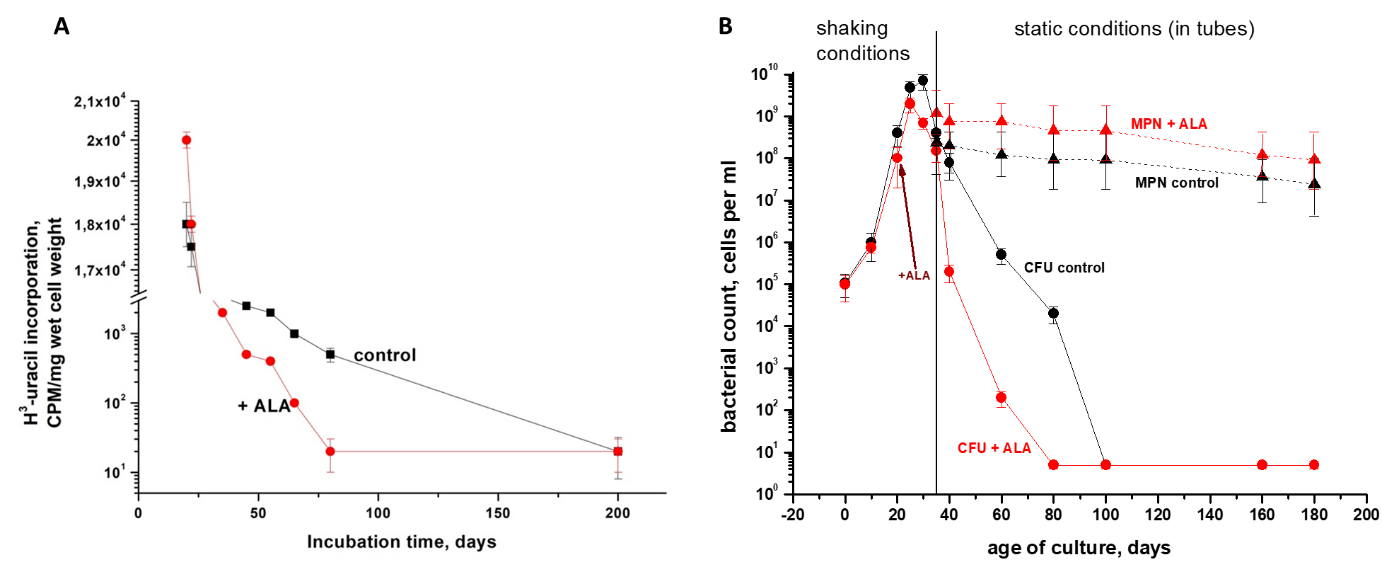


**Figure S1. Dynamics of changes in the rate of incorporation of H^3^ – uracil (A) and bacterial count (B) during the formation of dormant *M. tuberculosis* forms grown in the presence of and without 3 mM 5-aminolevulinic acid.** *Mtb* cells were inoculated in modified Sauton’s medium (see M&M), with initial pH 6.0, at a concentration of 10^5^ cells per ml, followed by incubation at 37 °C, under agitation of 200 rpm. Periodically, samples were withdrawn for estimation of the rate of H^3^-uracil incorporation (CPM) and bacterial count. Bacterial count was estimated on standard solid medium (CFU) and in special liquid medium (MPN assay) – see M&M section. (A) Squares - without ALA adding. Circles - ALA (3 mM) was added after 20 d post inoculation. (B) Circles - CFU. Triangles – MPN. Black – control without ALA adding. Red - ALA (3 mM) was added after 20 d post inoculation. The experiments were repeated three times, and a representative result is shown. MPN method was performed for two biological replicates in every experiment. For H^3^ – uracil incorporation and CFU bars demonstrate SD, for MPN bars demonstrate (95%) confidence limits.


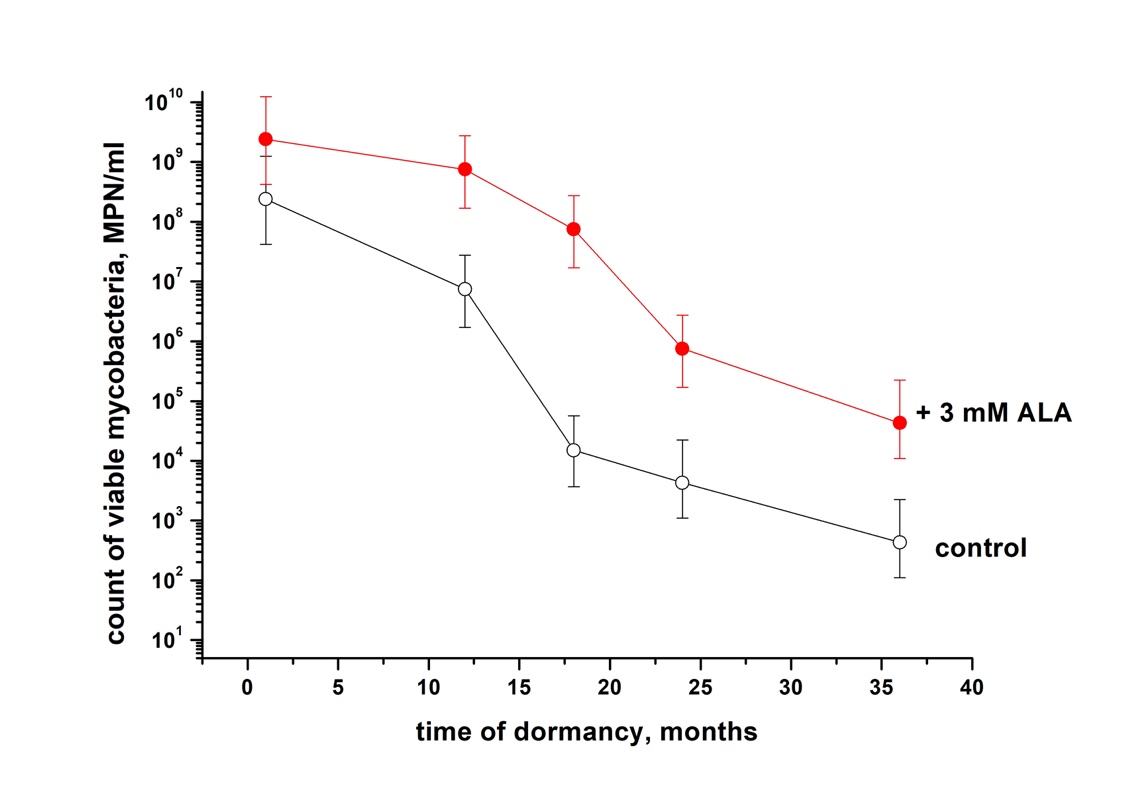


**Figure S2. Survival of *M. tuberculosis* cells during a period of long dormancy.** *Mtb* dormant cells obtained in the presence of or without 3 mM ALA in the modified Sauton’s medium were kept statically at room temperature. Periodically, samples were collected for viable bacterium estimation (MPN). The first time point corresponds to the point of 60 days shown on Figure S1b. For time 12 month and further CFUs were zero for every variant. Experiment was repeated twice, one representative experiment is shown. MPN method was performed for two biological replicates in every experiment. Bars represent (95%) confidence limits for the MPN assay.

**
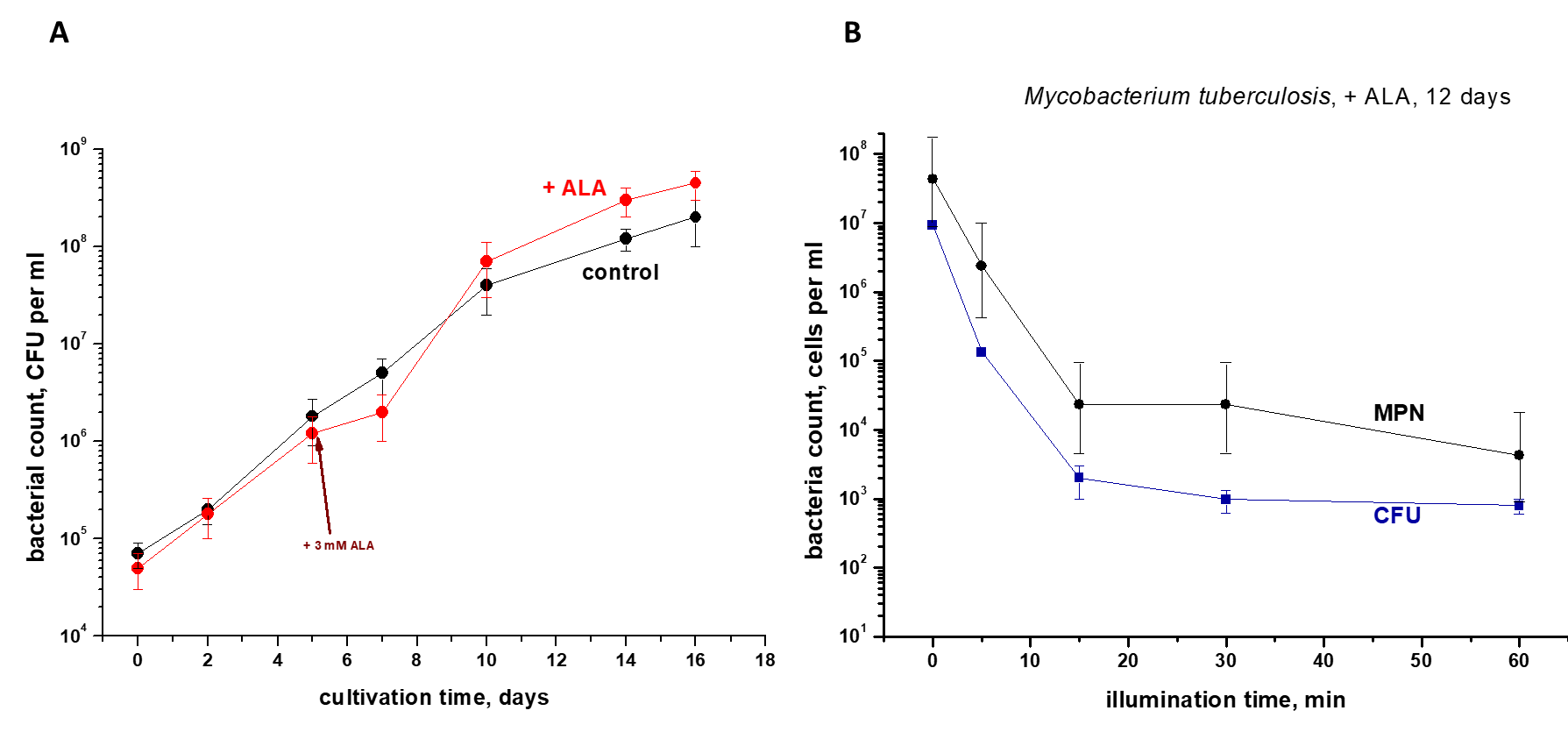
**

**Figure S3. Dynamics of changes in the growth rate of vegetative *M. tuberculosis* in the presence of and without 3 mM 5-aminolevulinic acid (A) and estimation of bacterial count of *M. tuberculosis* grown in presence ALA after illumination by CFU and MPN assays (B).** *Mtb* cells were cultivated in Middlebrook medium (Himedia, India) supplemented by ADC and 0.05 % of Tween-80 with the addition of 3 mM 5-aminolevulinic acid. Vegetative *Mtb* cells were subjected to PDI as described in M&M, at a different time of illumination at 565/24 nm, under static conditions. After exposure, cell viability was estimated by CFU and MPN assay. For CFU bars demonstrate SD, for MPN bars demonstrate (95%) confidence limits. MPN method was performed for two biological replicates in every experiment.

**
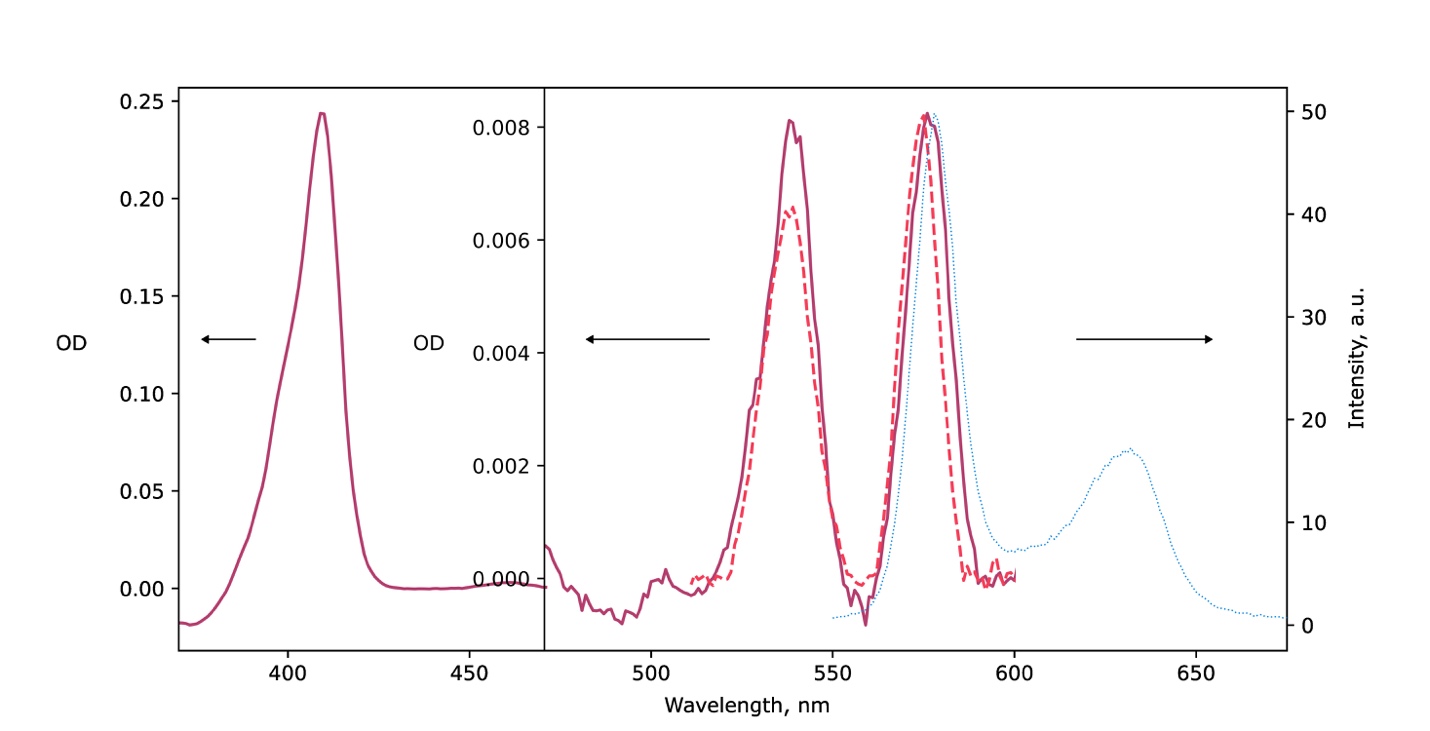
**

**Figure S4. Absorption (^___^) and Fluorescence (…) spectra of pure samples of Zn-coproporphyrin I and difference in absorption between spectra of extract and pure coproporphyrin (- - -) in chlorophorm/methanol/water.**

**
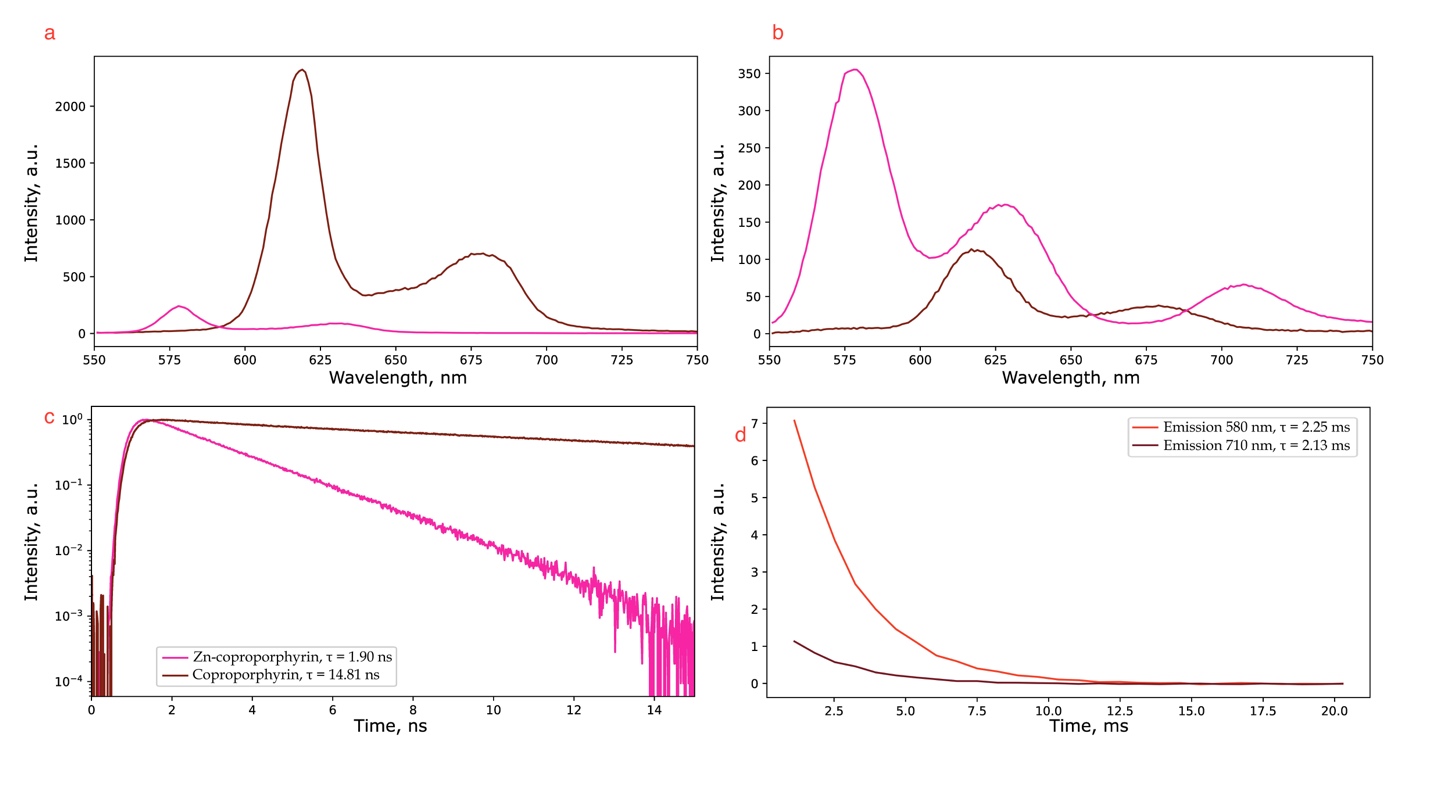
**

**Figure S5. (a) Fluorescence and (b) phosphorescence spectra of the pure samples of Zn-coproporphyrin and free coproporphyrin in 2% Triton X100 normalized to excitation optical density and (c) fluorescent life-time respectively. (D) Phosphorescence at 710 nm and delayed fluorescence at 580 nm lifetimes of the triton X100 extract of the dormant cells.**


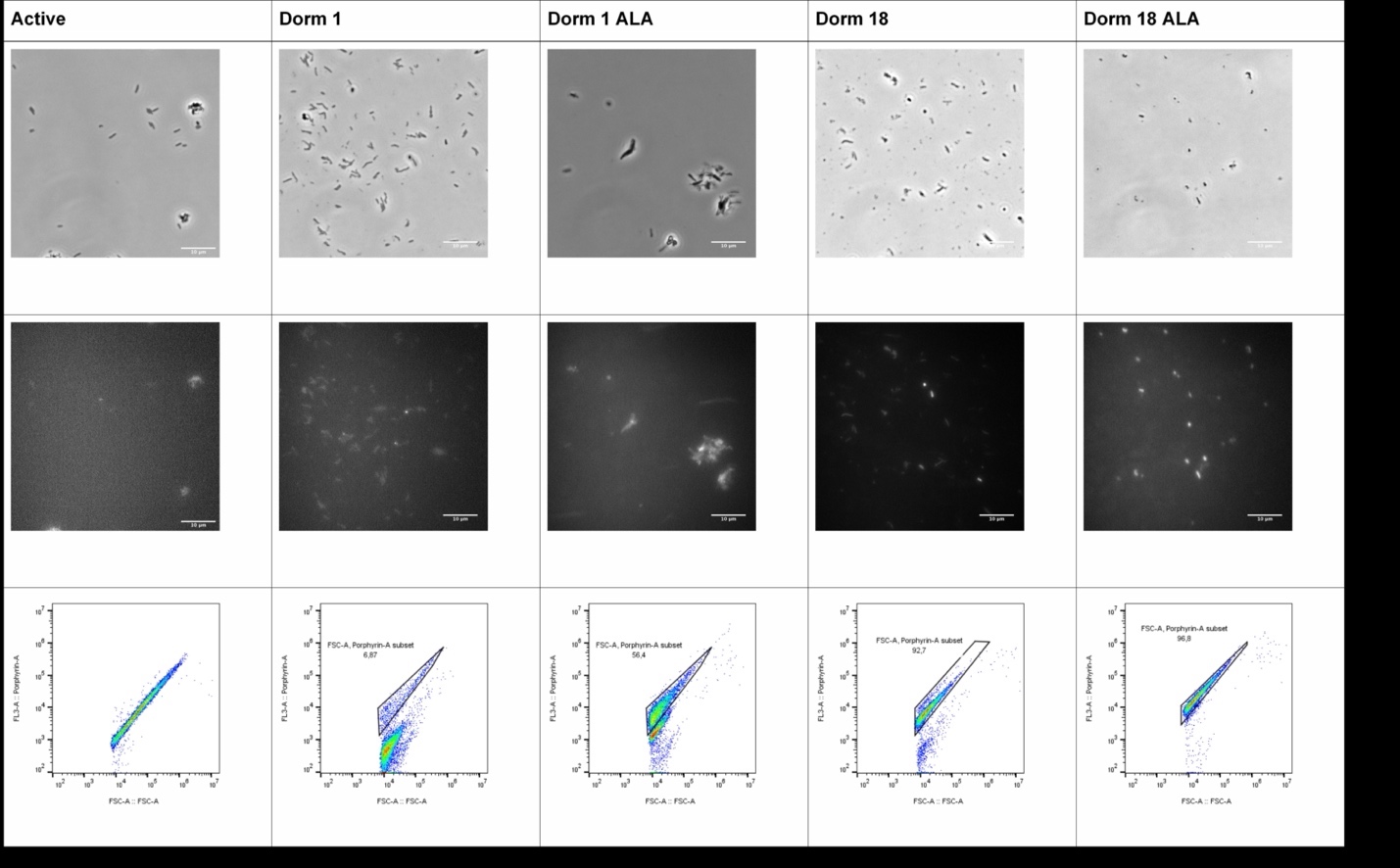


**Figure S6. Accumulation of a fluorescent-bright pigment in the cell envelope of the dormant *M. tuberculosis* cells.** Active – vegetative bacteria grown 10 d post inoculation under optimal conditions. Dorm1 – dormant bacteria 1-month-old. Dorm 1 ALA - dormant bacteria 1-month-old initially were grown in the presence of 3 mM ALA. Dorm18 – dormant bacteria 18-months-old. Dorm 18 ALA - dormant bacteria 18-months-old initially were grown in the presence of 3 mM ALA. Samples were prepared as described in M&M section.

Upper line - Phase-contrast and fluorescence microscopy of *Mtb* cells (magnification ×1500). Middle line - Epifluorescence microscopy was carried out in the ‘TRITC’ channel. The bar in each photo corresponds to 10 μm. Lower line - Flow cytometry analysis of auto-fluorescence of *Mtb* cells described in M&M. Voltage level for fluorescent PMT (659 V) was chosen to separate contribution of endogenous porphyrin fluorescence.

**
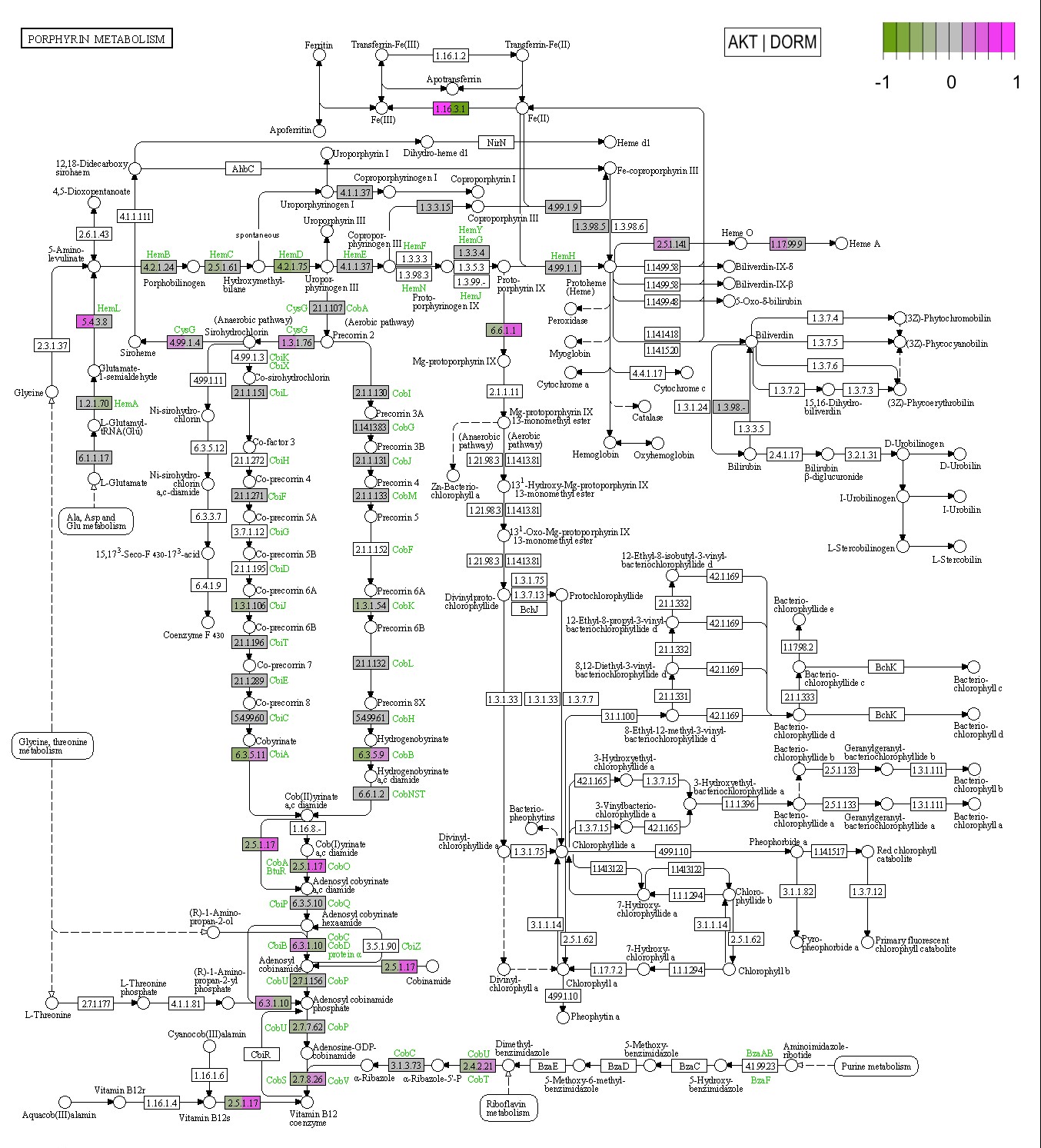
**

**Figure S7. Metabolic pathways of porphyrin metabolism in *M. tuberculosis*.** Comparison of actively growing cells with cells transitioning into a dormant state. A colour scale was plotted on the KEGG map, corresponding to the Z-score of changes in the level of gene expression. Genes not found in *M. tuberculosis* are not shaded. KEGG database was applied according to: Kanehisa, M., Furumichi, M., Sato, Y., Kawashima, M. and Ishiguro-Watanabe, M.; KEGG for taxonomy-based analysis of pathways and genomes. Nucleic Acids Res. 51, D587-D592 (2023)


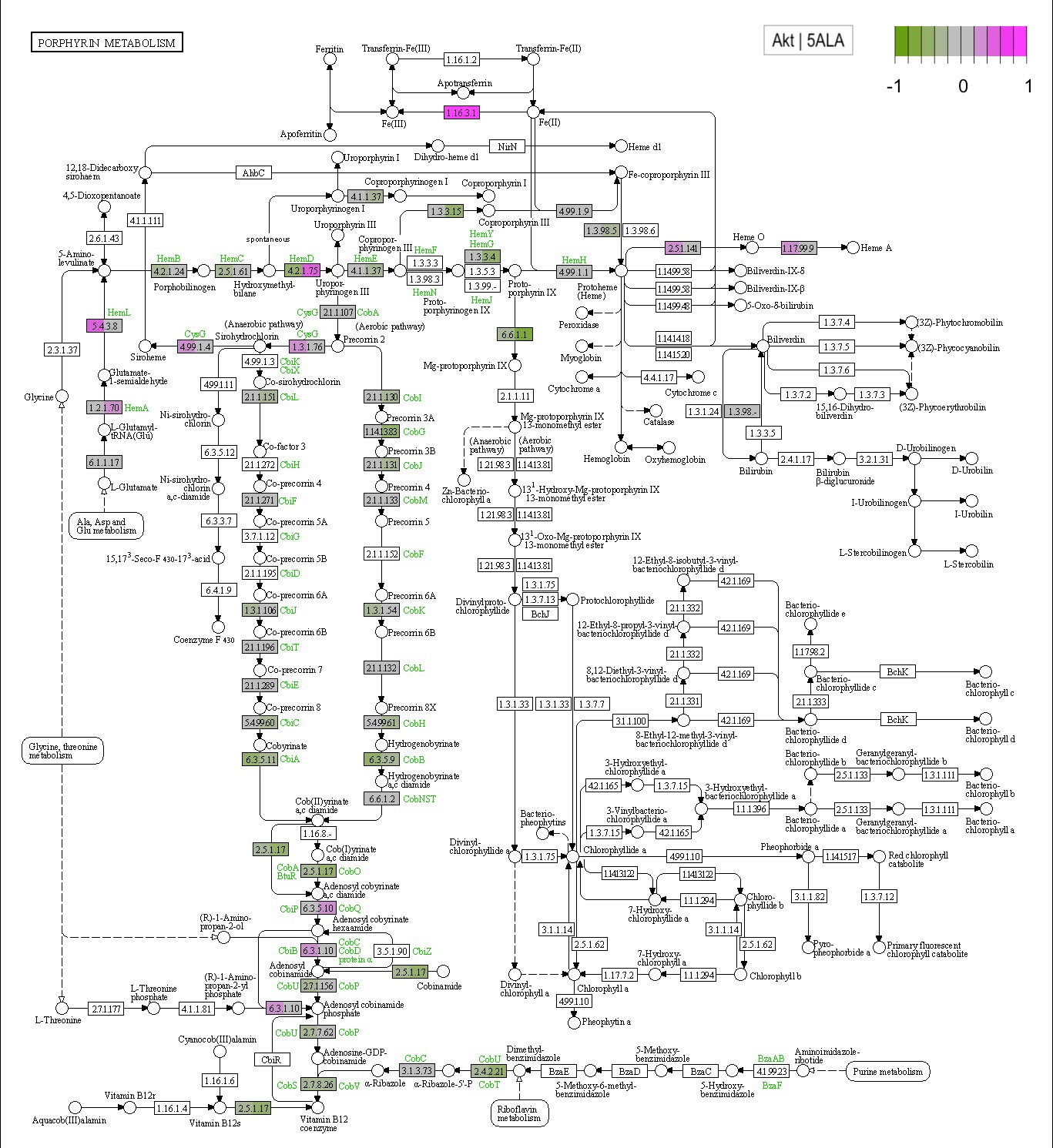


**Figure S8. Metabolic pathways of porphyrin metabolism in *M. tuberculosis*. Comparison of actively growing cells with cells treated with 5-aminolevulinic acid (5-ALA).** A colour scale was plotted on the KEGG map, corresponding to the Z-score of changes in the level of gene expression. Genes not found in *M. tuberculosis* are not shaded. KEGG database was applied according to: Kanehisa, M., Furumichi, M., Sato, Y., Kawashima, M. and Ishiguro-Watanabe, M.; KEGG for taxonomy-based analysis of pathways and genomes. Nucleic Acids Res. 51, D587-D592 (2023)


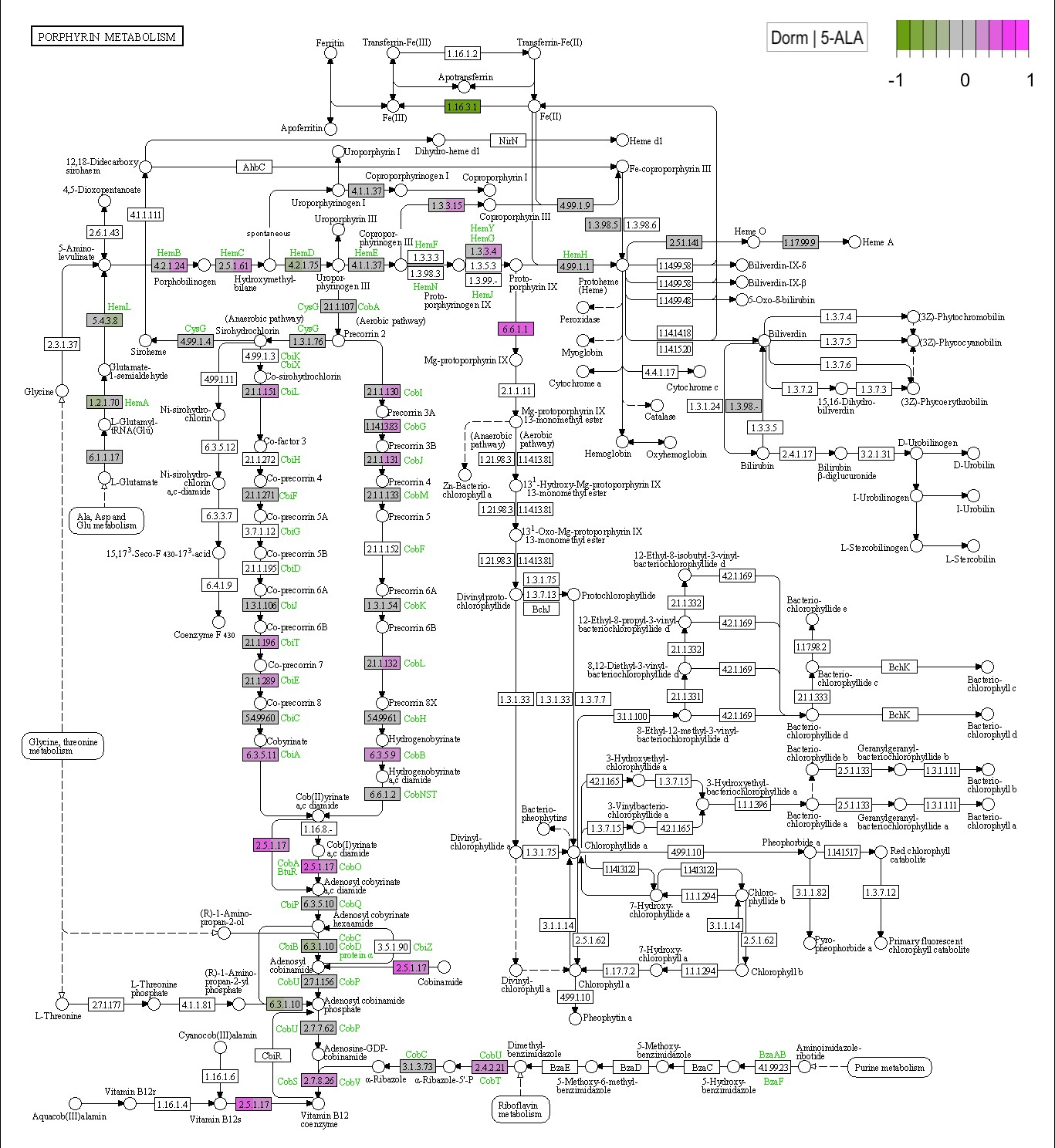


**Figure S9. Metabolic pathways of porphyrin metabolism in *M. tuberculosis*. Comparison of cells entering a dormant state with similar cells treated with 5-aminolevulinic acid (ALA).** A color scale was plotted on the KEGG map, corresponding to the Z-score of changes in the level of gene expression. Genes not found in *M. tuberculosis* are not shaded. KEGG database was applied according to: Kanehisa, M., Furumichi, M., Sato, Y., Kawashima, M. and Ishiguro-Watanabe, M.; KEGG for taxonomy-based analysis of pathways and genomes. Nucleic Acids Res. 51, D587-D592 (2023)

**Table S1. The content of porphyrins in vegetative and dormant *M. tuberculosis* cells grown in the presence of 5-aminolevulinic acid and without it (LC-MS analysis). According to the absorption spectra of the extracts (Figure 4), total fraction of Zn porphyrins may reach 82% for dormant cells and 72% for active cells with ALA.**

| ***Mtb* cell type** | **ng/mg cell wet weight** | | |
| --- | --- | --- | --- |
|  | **Coproporphyrin III (CP)** | **Uroporphyrin III** | **Coproporphyrin tetra-methyl ester** |
| **dormant** | 0 | 0,1± 0,04 | 0,12 ± 0,02 |
| **dormant + ALA** | 3,85 ± 0,021 | 1,7± 0,02 | 11,31± 0,05 |
| **vegetative** | 0 | 0,033 ± 0,01 | 0 |
| **vegetative + ALA** | 1,65 ± 0,12 | 2,48 ± 0,19 | 1,42 ± 0,1 |

**Table S2. The content of porphyrins in vegetative and dormant *M. tuberculosis* cells grown in the presence of 5-aminolevulinic acid (HR-MS analysis).**

| **Compound** | **Formula** | **Ion Type** | **Measured m/z** | **Theoretical m/z** | **∆m/z, ppm** |
| --- | --- | --- | --- | --- | --- |
| **Vegetative** ***M. tuberculosis* cells** |  |  |  |  |  |
| aceton-CH_3_OH extraction |  |  |  |  |  |
| Coproporphyrin | C_36_H_38_N_4_O_8_ | [M−2H]^2−^, M+1 | 326.6264 | 326.6288 | −7.3 |
| Zinc coproporphyrin | C_36_H_36_N_4_O_8_Zn | [M−2H]^2−^, M+2 | 358.0817 | 358.0831 | −3.9 |
| Trimethylcoproporphyrin | C_39_H_44_N_4_O_8_ | [M−H]^−^, M | 695.3049 | 695.3086 | −5.3 |
| Tetramethylcoproporphyrin | C_40_H_46_N_4_O_8_ | [M+H]^+^, M | 711.3353 | 711.3388 | −4.9 |
| chloroform-CH_3_OH-water extraction |  |  |  |  |  |
| Zinc uroporphyrin | C_40_H_36_N_4_O_16_Zn | [M−2H]^2−^, M | 445.0638 | 445.0636 | 0.4 |
| **Dormant** ***M. tuberculosis* cells** |  |  |  |  |  |
| aceton-CH_3_OH extraction |  |  |  |  |  |
| Trimethylcoproporphyrin | C_39_H_44_N_4_O_8_ | [M−H]^−^, M | 695.3094 | 695.3086 | 1.2 |
| Tetramethylcoproporphyrin | C_40_H_46_N_4_O_8_ | [M+H]^+^, M | 711.3367 | 711.3388 | −3.0 |
| Zinc Tetramethylcoproporphyrin | C_40_H_44_N_4_O_8_Zn | [M+H]^+^, M | 773.2504 | 773.2523 | −2.5 |
| chloroform-CH_3_OH-water extraction |  |  |  |  |  |
| Tetramethylcoproporphyrin | C_40_H_46_N_4_O_8_ | [M+H]^+^, M | 711.3370 | 711.3388 | −2.5 |
| Zinc Tetramethylcoproporphyrin | C_40_H_44_N_4_O_8_Zn | [M+H]^+^, M | 773.2504 | 773.2523 | −2.5 |

**Table S3. Statistically significant changes in the level of gene expression (up-regulation) of *M. tuberculosis* cells in various physiological states in the presence/absence of ALA.**

| **Up-regulated genes in dormant cells vs active cells, , FC >2** | | | | |
| --- | --- | --- | --- | --- |
| **Gene,ID** | **Gene** | **Functional,Category** | **Product** | **log2FC dorm_act** |
| Rv0062 | celA1 | intermediary metabolism and respiration | Possible cellulase CelA1 (endoglucanase)  (endo-1,4-beta-glucanase) (FI-cmcase) (carboxymethyl cellulase) | 1,1 |
| Rv0098 | fcoT | lipid metabolism | Probable fatty acyl CoA thioesterase type III FcoT | 1,24 |
| Rv0099 | fadD10 | lipid metabolism | Possible fatty-acid-CoA ligase FadD10  (fatty-acid-CoA synthetase) (fatty-acid-CoA synthase) | 1,28 |
| Rv0100 |  | conserved hypotheticals | Conserved hypothetical protein | 1,68 |
| Rv0101 | nrp | lipid metabolism | Probable peptide synthetase Nrp (peptide synthase) | 1,6 |
| Rv0158 |  | regulatory proteins | Probable transcriptional regulatory protein (possibly TetR-family) | 1 |
| Rv0188 |  | cell wall and cell processes | Probable conserved transmembrane protein | 1,71 |
| Rv0244c | fadE5 | lipid metabolism | Probable acyl-CoA dehydrogenase FadE5 | 1,38 |
| Rv0251c | hsp | virulence, detoxification, adaptation | Heat shock protein Hsp  (heat-stress-induced ribosome-binding protein A) | 1,53 |
| Rv0261c | rK3 | cell wall and cell processes | Probable integral membrane nitrite extrusion protein NarK3  (nitrite facilitator) | 1,37 |
| Rv0277c | vapC25 | virulence, detoxification, adaptation | Possible toxin VapC25, Contains PIN domain, | 1,55 |
| Rv0302 |  | regulatory proteins | Probable transcriptional regulatory protein  (probably TetR/AcrR-family) | 1,42 |
| Rv0303 |  | intermediary metabolism and respiration | Probable dehydrogenase/reductase | 1,38 |
| Rv0327c | cyp135A1 | intermediary metabolism and respiration | Possible cytochrome P450 135A1 Cyp135A1 | 1,48 |
| Rv0331 |  | intermediary metabolism and respiration | Possible dehydrogenase/reductase | 1,05 |
| Rv0341 | iniB | cell wall and cell processes | Isoniazid inductible gene protein IniB | 3,51 |
| Rv0342 | iniA | cell wall and cell processes | Isoniazid inductible gene protein IniA | 2,07 |
| Rv0343 | iniC | cell wall and cell processes | Isoniazid inductible gene protein IniC | 2,16 |
| Rv0350 | d K | virulence, detoxification, adaptation | Probable chaperone protein DnaK  (heat shock protein 70) (heat shock 70 kDa protein) (HSP70) | 1,09 |
| Rv0366c |  | conserved hypotheticals | Conserved hypothetical protein | 1,18 |
| Rv0421c |  | conserved hypotheticals | Conserved hypothetical protein | 1,07 |
| Rv0456A | mazF1 | virulence, detoxification, adaptation | Possible toxin MazF1 | 1,06 |
| Rv0478 | deoC | intermediary metabolism and respiration | Probable deoxyribose-phosphate aldolase DeoC  (phosphodeoxyriboaldolase) (deoxyriboaldolase) | 1,26 |
| Rv0481c |  | conserved hypotheticals | Hypothetical protein | 1,2 |
| Rv0498 |  | conserved hypotheticals | Conserved hypothetical protein | 1,2 |
| Rv0499 |  | conserved hypotheticals | Conserved hypothetical protein | 1,46 |
| Rv0542c | menE | intermediary metabolism and respiration | Possible O-succinylbenzoic acid--CoA ligase MenE  (OSB-CoA synthetase) (O-succinylbenzoate-CoA synthase) | 1,42 |
| Rv0563 | htpX | virulence, detoxification, adaptation | Probable protease transmembrane protein heat shock protein  HtpX | 1,12 |
| Rv0591 | mce2C | virulence, detoxification, adaptation | Mce-family protein Mce2C | 1,23 |
| Rv0725c |  | conserved hypotheticals | Conserved hypothetical protein | 1,16 |
| Rv0790c |  | conserved hypotheticals | Hypothetical protein | 1,51 |
| Rv0791c |  | conserved hypotheticals | Conserved protein | 1,89 |
| Rv0792c |  | regulatory proteins | Probable transcriptional regulatory protein  (probably GntR-family) | 2,11 |
| Rv0826 |  | conserved hypotheticals | Conserved hypothetical protein | 2,26 |
| Rv0837c |  | conserved hypotheticals | Hypothetical protein | 1,13 |
| Rv0847 | lpqS | cell wall and cell processes | Probable lipoprotein LpqS | 1,1 |
| Rv0960 | vapC9 | virulence, detoxification, adaptation | Possible toxin VapC9 | 1,23 |
| Rv0975c | fadE13 | lipid metabolism | Probable acyl-CoA dehydrogenase FadE13 | 1 |
| Rv0976c |  | conserved hypotheticals | Conserved hypothetical protein | 1,44 |
| Rv0989c | grcC2 | intermediary metabolism and respiration | Probable polyprenyl-diphosphate synthase GrcC2  (polyprenyl pyrophosphate synthetase) | 1,06 |
| Rv1026 |  | virulence, detoxification, adaptation | Conserved protein | 1,17 |
| Rv1032c | trcS | regulatory proteins | Two component sensor histidine kinase TrcS | 1,03 |
| Rv1033c | trcR | regulatory proteins | Two component transcriptional regulator TrcR | 1,03 |
| Rv1043c |  | conserved hypotheticals | Conserved hypothetical protein | 1,24 |
| Rv1048c |  | conserved hypotheticals | Hypothetical protein | 1,09 |
| Rv1073 |  | conserved hypotheticals | Conserved hypothetical protein | 1,13 |
| Rv1129c |  | regulatory proteins | Probable transcriptional regulator protein | 1,94 |
| Rv1130 | prpD | intermediary metabolism and respiration | Possible methylcitrate dehydratase PrpD | 3,47 |
| Rv1131 | prpC | intermediary metabolism and respiration | Probable methylcitrate synthase PrpC | 3,82 |
| Rv1142c | echA10 | lipid metabolism | Probable enoyl-CoA hydratase EchA10  (enoyl hydrase) (unsaturated acyl-CoA hydratase) (crotonase) | 1,62 |
| Rv1214c | PE14 | PE/PPE | PE family protein PE14 | 1 |
| Rv1256c | cyp130 | intermediary metabolism and respiration | Probable cytochrome P450 130 Cyp130 | 1,4 |
| Rv1396c | PE_PGRS25 | PE/PPE | PE-PGRS family protein PE_PGRS25 | 1,73 |
| Rv1402 | priA | information pathways | Putative primosomal protein N' PriA (replication factor Y) | 1,28 |
| Rv1471 | trxB1 | intermediary metabolism and respiration | Probable thioredoxin TrxB1 | 1,7 |
| Rv1480 |  | conserved hypotheticals | Conserved protein | 1,02 |
| Rv1481 |  | cell wall and cell processes | Probable membrane protein | 1,26 |
| Rv1497 | lipL | intermediary metabolism and respiration | Probable esterase LipL | 1,7 |
| Rv1514c |  | conserved hypotheticals | Conserved hypothetical protein | 1,11 |
| Rv1519 |  | conserved hypotheticals | Conserved hypothetical protein | 1,42 |
| Rv1532c |  | conserved hypotheticals | Conserved hypothetical protein | 1,06 |
| Rv1533 |  | conserved hypotheticals | Conserved protein | 1,04 |
| Rv1553 | frdB | intermediary metabolism and respiration | Probable fumarate reductase [iron-sulfur subunit]  FrdB (fumarate dehydrogenase) (fumaric hydrogenase) | 1,2 |
| Rv1561 | vapC11 | virulence, detoxification, adaptation | Possible toxin VapC11 | 1,08 |
| Rv1571 |  | conserved hypotheticals | Conserved protein | 1,16 |
| Rv1573 |  | insertion seqs and phages | Probable PhiRv1 phage protein | 1,62 |
| Rv1585c |  | insertion seqs and phages | Possible phage PhiRv1 protein | 1,38 |
| Rv1586c |  | insertion seqs and phages | Probable PhiRv1 integrase | 1,44 |
| Rv1708 |  | cell wall and cell processes | Putative initiation inhibitor protein | 1,03 |
| Rv1709 | scpA | cell wall and cell processes | Possible segregation and condensation protein ScpA | 1,43 |
| Rv1714 |  | intermediary metabolism and respiration | Probable oxidoreductase | 2,36 |
| Rv1715 | fadB3 | lipid metabolism | Probable 3-hydroxybutyryl-CoA dehydrogenase FadB3  (beta-hydroxybutyryl-CoA dehydrogenase) (BHBD) | 2,51 |
| Rv1716 |  | conserved hypotheticals | Conserved hypothetical protein | 2,1 |
| Rv1717 |  | conserved hypotheticals | Conserved hypothetical protein | 1,21 |
| Rv1767 |  | conserved hypotheticals | Conserved protein | 1,15 |
| Rv1808 | PPE32 | PE/PPE | PPE family protein PPE32 | 1,06 |
| Rv1809 | PPE33 | PE/PPE | PPE family protein PPE33 | 1,19 |
| Rv1816 |  | regulatory proteins | Possible transcriptional regulatory protein | 1,36 |
| Rv1835c |  | conserved hypotheticals | Conserved hypothetical protein | 1,06 |
| Rv1856c |  | intermediary metabolism and respiration | Possible oxidoreductase | 1,53 |
| Rv1870c |  | conserved hypotheticals | Conserved hypothetical protein | 1,12 |
| Rv1884c | rpfC | cell wall and cell processes | Probable resuscitation-promoting factor RpfC | 1,06 |
| Rv1959c | parE1 | virulence, detoxification, adaptation | Possible toxin ParE1 | 1,04 |
| Rv2016 |  | conserved hypotheticals | Hypothetical protein | 1,12 |
| Rv2017 |  | regulatory proteins | Transcriptional regulatory protein | 1,01 |
| Rv2052c |  | conserved hypotheticals | Conserved protein | 2,26 |
| Rv2053c | fxsA | cell wall and cell processes | Probable transmembrane protein FxsA | 1,99 |
| Rv2092c | helY | information pathways | ATP-dependent DNA helicase HelY | 1,2 |
| Rv2103c | vapC37 | virulence, detoxification, adaptation | Possible toxin VapC37, Contains PIN domain, | 1,1 |
| Rv2146c |  | cell wall and cell processes | Possible conserved transmembrane protein | 1,02 |
| Rv2225 | panB | intermediary metabolism and respiration | 3-methyl-2-oxobutanoate hydroxymethyltransferase PanB | 1,04 |
| Rv2249c | glpD1 | intermediary metabolism and respiration | Probable glycerol-3-phosphate dehydrogenase GlpD1 | 1,07 |
| Rv2253 |  | cell wall and cell processes | Possible secreted unknown protein | 1,51 |
| Rv2324 |  | regulatory proteins | Probable transcriptional regulatory protein (probably AsnC-family) | 1,12 |
| Rv2369c |  | conserved hypotheticals | Hypothetical protein | 1,57 |
| Rv2377c |  | lipid metabolism | Putative conserved protein MbtH | 2,01 |
| Rv2378c | mbtG | lipid metabolism | Lysine-N-oxygenase MbtG (L-lysine 6-monooxygenase)  (lysine N6-hydroxylase) | 1,57 |
| Rv2379c | mbtF | lipid metabolism | Peptide synthetase MbtF (peptide synthase) | 1,71 |
| Rv2380c | mbtE | lipid metabolism | Peptide synthetase MbtE (peptide synthase) | 1,87 |
| Rv2381c | mbtD | lipid metabolism | Polyketide synthetase MbtD (polyketide synthase) | 2,1 |
| Rv2382c | mbtC | lipid metabolism | Polyketide synthetase MbtC (polyketide synthase) | 2,12 |
| Rv2383c | mbtB | lipid metabolism | Phenyloxazoline synthase MbtB (phenyloxazoline synthetase) | 1,85 |
| Rv2386c | mbtI | lipid metabolism | Isochorismate synthase MbtI | 2,05 |
| Rv2450c | rpfE | cell wall and cell processes | Probable resuscitation-promoting factor RpfE | 1,1 |
| Rv2530c | vapC39 | virulence, detoxification, adaptation | Possible toxin VapC39, Contains PIN domain, | 1,07 |
| Rv2552c | aroE | intermediary metabolism and respiration | Probable shikimate 5-dehydrogenase AroE  (5-dehydroshikimate reductase) | 1,18 |
| Rv2592c | ruvB | information pathways | Probable holliday junction DNA helicase RuvB | 1,17 |
| Rv2593c | ruvA | information pathways | Probable holliday junction DNA helicase RuvA | 1,16 |
| Rv2594c | ruvC | information pathways | Probable crossover junction endodeoxyribonuclease RuvC  (holliday junction nuclease) (holliday junction resolvase) | 1,01 |
| Rv2641 | cadI | conserved hypotheticals | Cadmium inducible protein CadI | 1,75 |
| Rv2642 |  | regulatory proteins | Possible transcriptional regulatory protein  (probably ArsR-family) | 1,84 |
| Rv2710 | sigB | information pathways | RNA polymerase sigma factor SigB | 1,08 |
| Rv2745c | clgR | regulatory proteins | Transcriptional regulatory protein ClgR | 1,99 |
| Rv2760c | vapB42 | virulence, detoxification, adaptation | Possible antitoxin VapB42 | 1,01 |
| Rv2913c |  | intermediary metabolism and respiration | Possible D-amino acid aminohydrolase (D-amino acid hydrolase) | 1,42 |
| Rv2942 | mmpL7 | cell wall and cell processes | Conserved transmembrane transport protein MmpL7 | 1,02 |
| Rv2966c |  | intermediary metabolism and respiration | Possible methyltransferase (methylase) | 1,11 |
| Rv2973c | recG | information pathways | Probable ATP-dependent DNA helicase RecG | 1,09 |
| Rv3027c |  | intermediary metabolism and respiration | GCN5-related N-acetyltransferase | 1,15 |
| Rv3037c |  | conserved hypotheticals | Conserved hypothetical protein | 1 |
| Rv3054c |  | conserved hypotheticals | Conserved hypothetical protein | 1,94 |
| Rv3098A |  | virulence, detoxification, adaptation | PemK-like protein | 1,01 |
| Rv3100c | smpB | virulence, detoxification, adaptation | Probable SSRA-binding protein SmpB | 1,07 |
| Rv3175 |  | intermediary metabolism and respiration | Possible amidase (aminohydrolase) | 1,29 |
| Rv3177 |  | virulence, detoxification, adaptation | Possible peroxidase (non-haem peroxidase) | 1,51 |
| Rv3183 |  | regulatory proteins | Possible transcriptional regulatory protein | 1,14 |
| Rv3247c | tmk | intermediary metabolism and respiration | Thymidylate kinase Tmk (dTMP kinase) (thymidylic acid kinase)  (TMPK) | 1,42 |
| Rv3269 |  | virulence, detoxification, adaptation | Conserved protein | 1,54 |
| Rv3270 | ctpC | cell wall and cell processes | Probable metal cation-transporting P-type ATPase C CtpC | 1,7 |
| Rv3308 | pmmB | intermediary metabolism and respiration | Probable phosphomannomutase PmmB  (phosphomannose mutase) | 1,13 |
| Rv3384c | vapC46 | virulence, detoxification, adaptation | Possible toxin VapC46, Contains PIN domain, | 1,18 |
| Rv3402c |  | cell wall and cell processes | Conserved hypothetical protein | 1,78 |
| Rv3429 | PPE59 | PE/PPE | PPE family protein PPE59 | 1,5 |
| Rv3445c | esxU | cell wall and cell processes | ESAT-6 like protein EsxU | 1,22 |
| Rv3452 | cut4 | cell wall and cell processes | Probable cutinase precursor Cut4 | 1,04 |
| Rv3508 | PE_PGRS54 | PE/PPE | PE-PGRS family protein PE_PGRS54 | 1,3 |
| Rv3517 |  | conserved hypotheticals | Conserved hypothetical protein | 1,01 |
| Rv3578 | arsB2 | cell wall and cell processes | Possible arsenical pump integral membrane protein ArsB2 | 1,18 |
| Rv3600c |  | conserved hypotheticals | Conserved protein | 1,05 |
| Rv3602c | panC | intermediary metabolism and respiration | Pantoate--beta-alanine ligase PanC (pantothenate synthetase)  (pantoate activating enzyme) | 1,14 |
| Rv3603c |  | conserved hypotheticals | Conserved hypothetical alanine and leucine rich protein | 1,09 |
| Rv3660c |  | virulence, detoxification, adaptation | Conserved hypothetical protein | 1,08 |
| Rv3706c |  | conserved hypotheticals | Conserved hypothetical proline rich protein | 1,38 |
| Rv3797 | fadE35 | lipid metabolism | Probable acyl-CoA dehydrogenase FadE35 | 1,55 |
| Rv3848 |  | cell wall and cell processes | Probable conserved transmembrane protein | 1,87 |
| Rv3862c | whiB6 | regulatory proteins | Possible transcriptional regulatory protein WhiB-like WhiB6 | 6,28 |
| Rv3863 |  | conserved hypotheticals | Unknown alanine rich protein | 1,45 |
| Rv3876 | espI | cell wall and cell processes | ESX-1 secretion-associated protein EspI,  Conserved proline and alanine rich protein, | 1,02 |
| Rv3878 | espJ | cell wall and cell processes | ESX-1 secretion-associated protein EspJ,  Conserved alanine rich protein, | 1,09 |
| Rv3879c | espK | cell wall and cell processes | ESX-1 secretion-associated protein EspK,  Alanine and proline rich protein, | 1,08 |
| Rv3880c | espL | cell wall and cell processes | ESX-1 secretion-associated protein EspL | 1,04 |
| Rv3881c | espB | cell wall and cell processes | Secreted ESX-1 substrate protein B, EspB,  Conserved alanine and glycine rich protein | 1,03 |
| Rv3899c |  | conserved hypotheticals | Conserved hypothetical protein | 1,03 |
| **Up-regulated genes in active cells +5-ALA vs active cells ,FC > 2** | | | | |
| **Gene,ID** | **Gene** | **Functional,Category** | **Product** | **log2FC**  **ala_aсt** |
| Rv0100 |  | conserved hypotheticals | Conserved hypothetical protein | 1 |
| Rv0260c |  | regulatory proteins | Possible transcriptional regulatory protein | 1,24 |
| Rv1030 | kdpB | cell wall and cell processes | Probable potassium-transporting P-type ATPase B chain KdpB  (potassium-translocating ATPase B chain)  (ATP phosphohydrolase [potassium-transporting] B chain)  (potassium binding and translocating subunit B) | 1,12 |
| Rv1031 | kdpC | cell wall and cell processes | Probable potassium-transporting ATPase C chain KdpC  (potassium-translocating ATPase C chain)  (ATP phosphohydrolase [potassium-transporting] C chain)  (potassium binding and translocating subunit C) | 1,05 |
| Rv1195 | PE13 | PE/PPE | PE family protein PE13 | 1,31 |
| Rv1553 | frdB | intermediary metabolism and respiration | Probable fumarate reductase [iron-sulfur subunit]  FrdB (fumarate dehydrogenase) (fumaric hydrogenase) | 1,29 |
| Rv2381c | mbtD | lipid metabolism | Polyketide synthetase MbtD (polyketide synthase) | 1,26 |
| Rv2382c | mbtC | lipid metabolism | Polyketide synthetase MbtC (polyketide synthase) | 1,13 |
| Rv3402c |  | cell wall and cell processes | Conserved hypothetical protein | 1,1 |
| **Up-regulated genes in dormant cells +5-ALA vs dormant cells , FC > 2** | | | | |
| **Gene,ID** | **Gene** | **Functional,Category** | **Product** | **log2FC**  **ala_dorm** |
| Rv1405c |  | intermediary metabolism and respiration | Putative methyltransferase | 1,18 |
